# State-of-the-Art on Wound Vitality Evaluation: A Systematic Review

**DOI:** 10.3390/ijms23136881

**Published:** 2022-06-21

**Authors:** Aniello Maiese, Alice Chiara Manetti, Naomi Iacoponi, Eleonora Mezzetti, Emanuela Turillazzi, Marco Di Paolo, Raffaele La Russa, Paola Frati, Vittorio Fineschi

**Affiliations:** 1 Department of Surgical, Medical and Molecular Pathology and Critical Care Medicine, Institute of Legal Medicine, University of Pisa, 56126 Pisa, Italy; aniello.maiese@unipi.it (A.M.); alicechiara812@gmail.com (A.C.M.); naomiiacoponi@gmail.com (N.I.); eleonora.mezzetti@gmail.com (E.M.); emanuela.turillazzi@unipi.it (E.T.); marco.dipaolo@unipi.it (M.D.P.); 2 Department of Clinical and Experimental Medicine, University of Foggia, 71122 Foggia, Italy; raffaele.larussa@unifg.it; 3 Department of Anatomical, Histological, Forensic and Orthopedic Sciences, Institute of Legal Medicine, Sapienza University of Rome, Viale Regina Elena 336, 00161 Rome, Italy; paola.frati@uniroma1.it

**Keywords:** vitality, wound, autopsy, histology, immunohistochemistry, protein quantification, ribonucleic acids

## Abstract

The vitality demonstration refers to determining if an injury has been caused ante- or post-mortem, while wound age means to evaluate how long a subject has survived after the infliction of an injury. Histology alone is not enough to prove the vitality of a lesion. Recently, immunohistochemistry, biochemistry, and molecular biology have been introduced in the field of lesions vitality and age demonstration. The study was conducted according to the preferred reporting items for systematic review (PRISMA) protocol. The search terms were “wound”, “lesion”, “vitality”, “evaluation”, “immunohistochemistry”, “proteins”, “electrolytes”, “mRNAs”, and “miRNAs” in the title, abstract, and keywords. This evaluation left 137 scientific papers. This review aimed to collect all the knowledge on vital wound demonstration and provide a temporal distribution of the methods currently available, in order to determine the age of lesions, thus helping forensic pathologists in finding a way through the tangled jungle of wound vitality evaluation.

## 1. Introduction

Lesion vitality demonstration is one of the most challenging topics in forensic pathology. It has an undeniable importance in judicial processes, in which it can subvert the reconstruction of an event and influence the judgment. The vitality demonstration refers to determining whether an injury has been caused ante- or post-mortem, while wound age means to evaluate how long a subject has survived after the infliction of an injury [1]. A complex combination of events (acute inflammation, hemorrhage, proliferation, and remodeling) occurs immediately after wounding of tissue to re-establish its integrity and functionality. In the case of skin lesions, the presence of macroscopically evident blood infiltration of soft tissues can reveal the vitality of a bruise, while the change in coloration can indicate the time of survival [2]. However, wound examination with the naked eye is obviously not a reliable method to prove the vitality of a lesion. Histological analysis, based on hematoxylin-eosin, along with other stains (e.g., Prussian blue stain, elastica-van Gieson stain, etc.), helps visualize the vital reactions in human tissues [3]. Red blood cells infiltration, inflammatory reactions, presence of fibroblasts, macrophages, and immigrating granulocytes, as well as tissue alterations, are the main histological findings for wound vitality evaluation. However, this method has some limitations, such as operator dependency and the presence of staining artifacts. Moreover, some studies have shown that these characteristics may be also present in skin that is not vitally injured [4]. For these reasons, histology alone is not enough to prove the vitality of a lesion. Recently, immunohistochemistry, biochemistry, and molecular biology have been introduced in the field of lesion vitality and age demonstration. Immunohistochemistry has been widely studied and gained increasing importance [4,5]. It is based on the immunological reaction between an antigen and antibody [6]. The most used immunostains highlight the presence of cytokines (IL-1, IL-6, TNF-α, etc.), inflammatory cells (mast cells, myofibroblast cells, etc.), and other damaged-related molecules in tissues [7,8,9]. Moreover, researchers found that some molecules’ expression varies in a time-dependent manner, so they can be used in wound age determination [10]. The significant limits of immunohistochemistry are operator dependency and the lack of a standardized and internationally accepted protocol. Researchers investigated different molecules, and there is no uniformity. In recent years, more unbiased methodologies have been sought. In this regard, of great interest is the analysis of electrolyte (sodium, potassium, cloridium, calcium, and magnesium) or protein (albumin, troponin, erythropoietin, etc.) concentration in biological fluids and tissues [11]. The changes in their concentration could depend on the presence of inflammatory processes, which means they can be used in differentiating vital or post-mortem damage. Moreover, electrolyte and protein quantification are not operator dependent and could be easily standardized [12]. On the other hand, there are problems concerning the collection and preparation of the samples. Moreover, biochemical studies are limited to the early post-mortem period (usually within 48 h after death) because the decomposition could alter their results [13]. Another possible tool in lesion vitality evaluation is genetic analysis. The messenger RNA (mRNAs) and microRNAs (miRNAs), which control the expression of various proteins involved in the inflammation processes, could be studied to evaluate their changes in wounded tissues [14,15,16]. Differential mRNAs and miRNAs expression in wounds over time has been demonstrated, making them hypothetically useful in determining the timing of a lesion [17,18]. Moreover, since miRNAs are implicated in the post-transcriptional protein regulation, their changes should be detectable earlier than changes in proteins concentration, supposedly allowing for the distinction between ante- and post-mortem wounds, even when the survival time is very short.

This review aimed to collect all the knowledge on vital wound demonstration and provide a temporal distribution of the methods currently available, in order to determine the age of lesions, thus helping forensic pathologists in finding a way through the tangled jungle of wound vitality evaluation.

## 2. Materials and Methods

The present systematic review was carried out according to the preferred reporting items for systematic review (PRISMA) standards [19]. Systematic literature search and critical review of the collected studies were conducted. An electronic search of PubMed, Science Direct Scopus, Google Scholar, and Excerpta Medica Database (EMBASE), from database inception to March 2022, was performed. The search terms were “wound”, “lesion”, “vitality”, “evaluation”, “immunohistochemistry”, “proteins”, “electrolytes”, “mRNAs”, and “miRNAs” in the title, abstract, and keywords. The bibliographies of all located papers were examined and cross-referenced, in order to further identify relevant literature. A methodological appraisal of each study was conducted according to the PRISMA standards, including an evaluation of bias. The data collection process included the study selection and data extraction. Two researchers (N.I. and E.M.) independently examined the papers with titles or abstracts that appeared to be relevant and selected those that analyzed wound vitality demonstration. Researchers resolved their disagreement concerning works eligibility by consensus. Only papers in English were included in the research. Three investigators performed data extraction (A.C.M., N.I., and E.M.), and two other investigators verified them (A.M., P.F.), which were again verified by two other investigators (E.T. and V.F.). This study was exempt from institutional review board approval, as it did not involve human subjects.

## 3. Results

**Figure 1:**
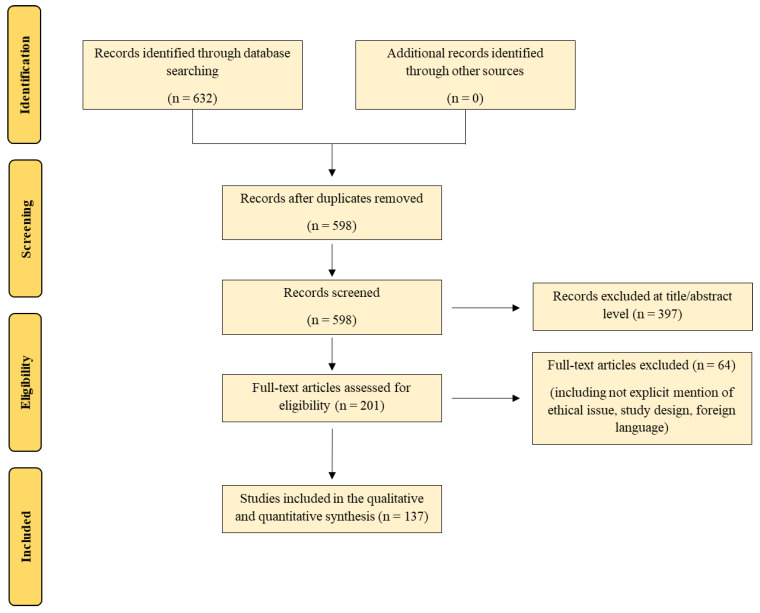
Our review strategy following PRISMA standards.

The search performed, as described above, identified 632 articles, which were screened to exclude duplicates. The resulting 598 reference lists were then screened based on their title and abstract, which left 201 articles for further consideration. Non-English papers were excluded. The following inclusion criteria were used: (1) original research articles, (2) communication, (3) case reports/series, and (4) review and mini-review. These publications were carefully evaluated, considering the main aims of the review. Review and mini-review have not been included in the qualitative synthesis, but they have been used to verify any missing paper. This evaluation left 137 scientific papers. Figure 1 illustrates our search strategy.

The papers resulting from our research have been divided into three groups: quantitative analysis in biological fluids and tissues of various markers (24 papers), immunohistochemistry (84 papers), and ribonucleic acids studies (20 papers on mRNAs, 21 papers on miRNAs). Table 1, Table 2 and Table 3 show a brief description of these three groups of studies, respectively.

The “quantitative analysis in biological fluids and tissues of various markers” group has been divided into two sub-groups, i.e., biological fluids and tissues, as well as the “ribonucleic acids” group, in order to simplify the fruition of the tables. To quantify proteins level in tissues, different types of analysis have been used, such as radioimmunoassay, photometric analysis, electrophoresis, matrix-assisted laser desorption/ionization-time-of-flight (MALDI-TOF), electrospray ionization-ion trap mass spectrometry (ESI-IT-MS), enzyme-linked immunosorbent assay (ELISA), nano-liquid chromatography (nano-LC) MS, and high-performance liquid chromatography (HPLC), while ions’ quantification are made by atomic absorption spectrophotometry.

Some studies included different kinds of analyses to investigate the differential protein expression in tissues; for example, He et al. applied both a Western blot analysis to quantify the protein levels and the real-time quantitative polymerase chain reaction (RT-qPCR) to evaluate the mRNA expression [40]. In such cases, we included the papers in both the relative tables, adding an asterisk near the reference (*). In the “miRNA” group, we included several papers aimed to investigate the role of specific miRNAs in wound healing and not the differential expression between vital and non-vital injuries. The reason for this choice is that miRNA studies in wound vitality demonstration are a novelty in this field, and only a few of them have been conducted on autoptic human samples. However, we wanted to collect the current knowledge on the role of miRNAs in wound healing, hoping that could be useful for further works in this field.

Considering all the analysis methods, the most investigated tissue is the skin, and only a few studies also evaluated the vitality reaction in other tissues (e.g., the skeletal muscle, brain, liver, kidney, etc.). The most studied method in wound vitality demonstration is immunohistochemistry (84/137 papers), and fibronectin is the most searched protein (15/85 papers).

## 4. Discussion

**Figure 2:**
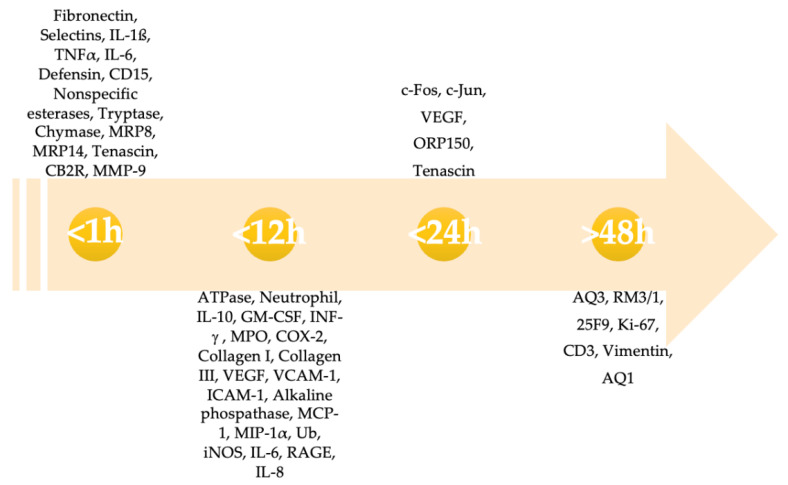
The timing of positivity of several immunohistochemical vitality markers after wounding. This figure includes exclusively markers for the skin. GM-CSF, granulocyte macrophage-colony stimulating factor; ICAM-1, intercellular adhesion molecule 1; IL, interleukin; iNOS, inducible nitric oxide synthase; MCP, monocyte chemoattractant protein; MMP, matrix metallopeptidase; RAGE, receptor for advanced glycation endproducts; TNF-α, tumor necrosis factor α; VEGF, vascular endothelial growth factor; VCAM-1, vascular cell adhesion molecule-1.

To determine if a lesion has been produced in life or post-mortem, as well as how much time has passed between the lesion’s production and death, is often crucial to understanding if there was a causal relationship between the wound and death. However, wound vitality evaluation is one of the most challenging fields for the forensic pathologist because there is not a standardized and internationally accepted method to prove the vitality of a lesion and its age. We performed a comprehensive review on wound vitality demonstration, in order to collect the current knowledge in this field. We found 137 papers that are heterogeneous and used different investigation methods; this is the greatest limitation of our work, which did not allow us to perform a proper quantitative synthesis. As the results of our review show, the most studied method for evaluating the vitality of a lesion in the literature is immunohistochemistry. It has been mainly used to highlight the presence of a healing and inflammatory response, which should be absent in post-mortem lesions. Nowadays, immunohistochemistry could be considered the “gold standard” in distinguishing between ante- and post-mortem lesions [113]. It is a morphological technique and could show the different distribution of the molecules of interest in the harmed tissue alongside their quantification. It is easily applicable, it does not require expensive or sophisticated machinery to be done, and it could also be applied in formalin-fixed paraffin embedded tissues, which means it is also possible to evaluate unsolved “cold cases” [157]. On the other hand, the quantification of protein expressions through immunohistochemical staining is highly operator-dependent, and the quality of staining can be influenced by many variables, so it could lead to errors or scarcely reproducible results [158,159]. Moreover, robust immunohistochemical protocols and automated measure procedures have not been developed yet in forensic pathology, as has been accomplished in clinical disciplines [160,161,162]. Besides, several immunohistochemical vitality markers have been tested. In Figure 2 and Table 4, we collected the most promising immunohistochemical vitality markers in skin, concerning the timing of positivity, as described in the collected papers.

Another field in wound vitality determination is represented by blood coagulation and hemostasis. Molecules involved in these processes are potentially valuable due to their early production in wounding and healing. Studies on FXIII, which regulates the cross-linking process of fibrin monomers and therefore clots stabilization, and its positive effects against MPPs action on fibroblast cultures could be encouraging. Moreover fibrin organization in the blood clot seems to be promising as a potential timing technique. It has been observed that, when using the Picro-Mallory staining method, fibrin stained either red, violet, or blue according to clot maturation timing. At 30 minutes to 6 h, fibrin stained red, from 6 to 12 h appeared purple or violet while in clots older than 24 h fibrin stained blue [122]. Fibrin deposits could also be found in vital bone fractures from 34 minutes to 26 days after wounding [81].

**Figure 3:**
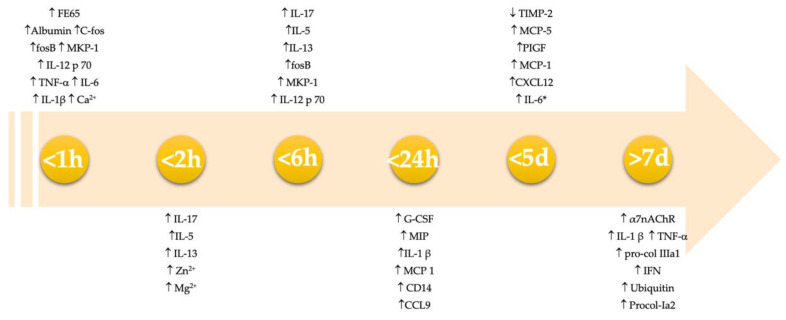
The differential expression of different biomarkers and electrolytes variations in skin as time (days) after wounding progress. H means “hours”; d means “day(s)”. We included all the biomarkers that showed a variation (increasing or decreasing) before 21 days. Regarding the biomarkers that showed an expression during an interval, only the upper limit has been considered. *IL 6 has been considered in different studies; it shows a different expression only in two studies.

Some researchers tried to apply quantitative techniques in lesion vitality evaluation, such as protein or ions quantification. In Figure 3, the main vitality markers that are evaluable with quantitative analysis of the skin are presented in a timeline. However, as our results show, there is only a minority of papers about this topic (24/137); therefore, these methodologies have not been sufficiently tested to be used in lesion vitality evaluation, especially when it is done for forensic purposes and these data need to be used in courts.

In Table 5, we collected both electrolytes and biomarkers as their concentration and expression variates in time after wounding, as described in the gathered papers. Electrolytes, such as nagnesium and calcium, are deeply involved in enzyme regulation and cell metabolism at the injured site, as well as in muscular contractility. Moreover, elevated levels of zinc can be found in later phases of reparation and seems to be involved in various inflammatory processes [23]. Sodium and potassium level variations could be related to membrane disruption, due to trauma, which causes a change in membrane potentials [21].

In recent years, the great development of genetic techniques has allowed for studying ribonucleic acid variations as vitality markers. One of the main problems in this field is represented by the immediate post-mortem period, a phase characterized by the persistence of vital reactions (so-called “residual-life phenomena”) within a very short time after death [163,164]. In fact, metabolic processes and vital activities do not cease in all the cells and tissues at the same time; so, immediately after death, they could mimic vital reactions, making it hard to differentiate vital from early post-mortem findings [165]. This problem involves a wide range of techniques used in wound vitality evaluation, from immunohistochemistry to miRNAs and proteomics. This limit of vitality markers could be addressed, when solving the single case, with careful evaluation of circumstantial data, case investigations, and medical history review. When confronted with an autopsy, forensic pathologists should always adopt a comprehensive approach, collaborating with the investigators.

Another issue in vitality demonstration is represented by post-mortem manipulations. It could happen that certain circumstances or post-mortem activities could artificially induce the findings that appear vital [166]. For example, ventilation could reproduce emphysema, and resuscitation maneuvers, which keep blood circulating, could induce post-mortem red blood extravasation in tissues.

Eventually, a limitation of the analyses described in this paper could be the decomposition and other post-mortem alterations (e.g., maceration or adipocere formation). Biomarkers and electrolytes, especially in fluids, are certainly influenced by the putrefactive phenomena and the reliability of their determination for wound vitality demonstration decreases when the post-mortem interval (PMI) increases. Immunohistochemistry seems reliable even when the corpse is putrefied, as some Authors demonstrated [53,67,85]. On the other hand, it is possible that decomposition, altering the tissues’ architecture, could influence not only the intensity of the immunohistochemical response, but also its localization. mRNAs and miRNAs are stable molecules and theoretically they could be less influenced by the putrefaction. However, there are not a lot of studies investigating the trustworthiness of these analyses in such conditions (we found only three papers evaluating immunohistochemistry in putrefied corpses) [53,67,85]. Therefore, more studies are needed to deepen the role of decomposition in the applicability of such techniques.

**Figure 4:**
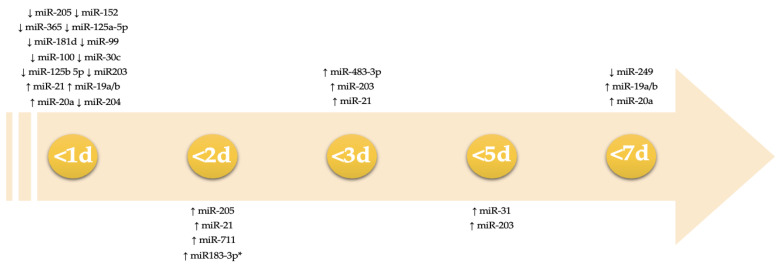
This figure shows the differential expression of miRNA in time (days) after wounding. We included only those miRNAs that showed a variation in expression, according to a precise timeframe. miRNAs variations that occurred within the first 24 h are summarized on the first day. *miR-183–3p shows different expressions in rats and humans; in rats, it was overexpressed within 120 h after wounding, whereas in humans, it was in 48 h. ↑ indicates up-regulation; ↓ down-regulation.

Since mRNAs and miRNAs have a role in protein production at a very early step, they could hypothetically be used as really precocious markers of an inflammatory response, differentiating vital and post-mortem lesions, even when the survival time is very short or such influencing factors occurred. Table 6 and Figure 4 show the timeline of detectability of some mRNAs and miRNAs implicated in wound healing, which may be used in lesion aging.

In the past few years, miRNAs have attracted researchers’ attention because they are involved in various inflammation and healing processes. Among the molecular pathways highlighted in this work, we found that miR-19a/b and miR-20a suppress poly(I:C)-induced expression of CXCL8, CXCL5, TNF-α, and IL-1A, proinflammatory chemokines, and cytokines at the mRNA level by regulating the nuclear factor kappa-light-chain-enhancer of the activated B cell (NF-кB) signaling pathway [156]. This pathway is activated by the tumor necrosis factor (TNF-α), which phosphorylates the inhibitor of nuclear factor kappa B (IкB). Similarly, miR-92a-3p, inhibits the intracellular transduction of the toll-like receptors (TLR), hence its role as a pro-inflammatory stimulus [149]. Moreover, miR-19b, targeting C-C motif chemokine ligand 1 (CCL1), seems to be involved in the regulation of the transforming growth factor-ß (TGF-ß) signaling pathway. This pathway, which is involved in all the phases of the repair process, is also targeted by miR-26a, miR-149, and miR-21 [153]. MiR-149, in particular, could be able to contain the inflammation process by downregulating the expression of IL-1α, IL-1ß, and IL-6 and is believed to act as a positive regulator of the skin healing process [145]. In corneal epithelial cells, miR-205 stimulates the healing process by inhibiting the inwardly rectifying K+ channel, KCNJ10 [141]. Furthermore, miR-205 is a positive regulator of keratinocytes migration, actively altering the organization of F-actin, decreasing cell-substrate adhesion, and, along with miR-184, regulating the SH2-containing phosphatidylinositol 3,4,5-trisphosphate 5-phosphatase (SHIP2) levels and phosphorous protein kinase B (AKT) signaling [134]. These miRNAs act in a very peculiar way; it seems that miR-184 can suppress the expression of miR205, therefore maintaining elevated SHIP2 levels. Additionally, SHIP2′s influence on keratinocyte migration could be both positive and negative, depending on the local levels of phosphoinositide pools, which are also involved in the cell adhesion process. Similarly, miR-205 downregulation determines the enhancement of cell migration by suppressing F-actin in HEKs and altering the levels of p-Akt, thus activating Rho, p-cofilin, and ERM. All these proteins are strictly associated with processes of remodeling and migration [167,168]. Members of the miR-99 family act as regulators in cell proliferation, apoptosis, and migration, through the PIK3/AKT pathway, therefore influencing the mTOR signaling pathway [140]. The mTOR pathway is also associated with the re-epithelialization of skin wounds [169]. Among this family, for example, miR-100 acts to reduce the phosphorylation of signaling molecules, such as p70 S6 kinase (p70S6K) and eukaryotic translation initiation factor 4E binding protein 1 (4E-BP1), which is involved in the pathway previously described. Another important pathway targeted by miRNAs, such as miR-149 and miR-222, is the MAPK pathway. MAPKs direct different cellular responses to stimuli including heat shock and gene expression regulation, cell proliferation, differentiation, and survival [142]. Furthermore miR-222 targets different genes, such as axin-like protein 2 (AXIN2), Dickkopf-related protein 2 (DDK2), and FRAT regulator of the Wnt signaling pathway 2 (FRAT2), therefore influencing the wingless-related integration site (Wnt) signal [169,170]. Pastar et al. found that miR-21 and miR-130a overexpression leads to inhibition of the epithelial growth factor (EGF) pathway by suppressing the early growth response 3 (EGR3) gene [137]. Suppression of EGR3 and vinculin could also be linked to the inhibition of keratinocyte migration in chronic wounds. Another important target of miR-21 is the leptin receptor (LepR) gene in the epidermis; this signaling is well-renowned as a pleiotropic stimulus on wound healing. MiR-203, as highlighted by Viticchiè et al., could be crucial in the regulation of different pathways, such as p63, LIM, and SH3 domain protein 1 (Lasp1), as well as the Ras-related nuclear protein (Ran) and Ras-associated and Pleckstrin homology domains 1 (Raph1), which are implicated in the re-epithelialization process [138]. The downregulation of this miRNA could probably mediate the switch to activated keratinocytes in wound closure; on the contrary, its overexpression could be the stimuli to commitment to differentiation in the healthy epidermis, as well as in injured ones. Furthermore, when miR-203 is upregulated, we can assist in an alteration of the Wnt/B-catenin signaling pathway, thus determining the decreased levels of MAPK8, MAPK9, Rho-associated coiled-coil-containing protein kinase 2 (ROCK2), and protein kinase C alpha (PRKCA). In addition, miR-203, through the IL8/AKT pathway, is implicated in the epithelial-mesenchymal transition (EMT) process [150]. MiRNAs are also involved in the angiogenesis process, namely miR-26a regulates the bone morphogenetic protein (BMP)/small mother against the decapentaplegic 1 protein (SMAD1) signaling pathway. The angiogenic impulse is carried out by BMP, as well as by the vascular endothelial growth factor (VEGF), which has a crucial role in cell migration and proliferation, in order to form new blood vessels [143]. Icli et al. found that miR-26a plays a part in promoting fibroblast migration in wound sites. miR-21 has multiple targets, such as Smad and Smad7, involved in promoting collagen deposition in granulation tissue [143]. Wang et al.’s results validate the involvement of this miRNA in both collagen deposition and wound contraction [139]. Table 7 shows a summary of some of the main molecular pathways influenced by miRNAs.

Even if miRNAs and genetic analyses were promising in wound vitality determination, there are not enough studies in this field, and a lot is still to be disclosed. It is not clear which confounding factors may influence their expression. Therefore, further studies are needed to allow for their use in everyday forensic practice. Furthermore, their determination is still limited to the quantitative technique. In our opinion, it is fundamental to develop a standardized approach that combines histological, immunohistochemical, and genetic methods. Only an interdisciplinary analysis would provide data reliable enough to be used in wound vitality demonstration for forensic purposes.

## 5. Conclusions

In the literature, several studies investigated the potential use of several markers in wound vitality demonstration [171,172,173,174,175,176,177]. However, a standardized method is not available yet, and these data could not be reliably used in forensic practice [178]. There are still several unknown factors that may influence the protein expression and molecular pathways involved in inflammation and wound healing, thus inducing misinterpretation [179,180,181]. In this review, we collected the current knowledge in wound vitality demonstration, through different fields of research; however, more evidence is certainly required. The need for an internationally accepted and interdisciplinary approach is urgent; we hope that, through this review, the readers find inspiration for further research, in order to deepen this topic.

## Figures and Tables

**Table 1:** The results of our review on vitality markers in biological fluids and tissues through quantitative analysis (24 articles). * These studies included different kinds of analyses, in order to investigate the differential protein expression in tissues, and they have been included in both the relative tables. α7nAChR indicates α7 nicotine acetylcholine receptor; ATF, activating transcription factors; CaMK II delta, calcium–calmodulin-dependent protein kinase II delta; CORT, corticosterone; CXC, keratinocytes-derived chemokine; CXCR, chemokine receptor; CRP, C-reactive protein; EPO, erythropoietin; HAX-1, HCLS1-associated protein X-1; HMGB1, high-mobility group box-1; IL-6, interleukin-6; LBP, lipopolysaccharide binding; LC3-II, lipid conjugated form II; LTB4, leukotriene B4; Mb, myoglobin; MI, myocardial infarction; MyoD, myoblast determination protein; MPO, myeloperoxidase; MT1-MMP, membrane type-1 matrix metalloproteinase; p62, sequestosome; Pax7, paired-box transcription factor 7; PCT, procalcitonin; RAGE, receptor for advanced glycation end products; sIL-2R, soluble interleukin-2 receptor; sTREM-1, soluble triggering receptors expressed on myeloid cells type 1; TIMP-2, tissue inhibitor of metalloproteinase-2; 1; VEGF, vascular endothelial grow factor; RT-PCR, real-time polymerase chain reaction.

**Biological Fluids**				
**References**	**Type of Paper**	**Model**	**Fluid**	**Brief Description**
Zhu et al. 2001 [20]	Original research	Human	Urine	The study aimed to investigate differential PM urinary Mb levels for determining the cause of death. PMI < 48 h did not influence urinary Mb levels, while PMI > 48 h showed increased levels (PM/putrefactive changes). Urinary Mb levels were increased when survival time was longer (>12–24 h, no linear correlation), as well as in some cases of vital muscle damage (e.g., fire fatalities, drowning, and head trauma), while they were not in cases of natural death due to MI. In cases of minor muscle damage (e.g., head traumas), the urinary Mb elevation was related to the survival time. The comparison between traumatic and non-traumatic deaths was not performed.
Quan et al. 2008 [11]	Original research	Human	Serum	Autoptic samples were analyzed (PMI tested < 48 h). EPO levels were increased in blunts produced 7 days after death, and its increase was higher in non-acute deaths due to wounds.
Amany Abdel-Raham et al. 2018 [21]	Original research	Animal	CSF	K+ was significantly higher in TBI than in controls (no traumatized animal) when the samples were collected 12 h after death (no statistical difference when PMI < 12 h). Na+ was significantly higher in controls than TBI, when the samples were collected at the time of death and 6 h after death. Ca^2+^ was significantly higher in TBI than controls, when the samples were collected at the time of death and 6 h after death, while it was higher in controls than TBI, when the PMI was 12 h; albumin was higher in TBI than controls only at the time of death (no statistical difference when PMI was 6 or 12 h). The total leucocytic count was significantly higher in TBI than controls, regardless to the PMI (PMI tested 0–12 h).
			Plasma	Uric acid and ammonia were significantly higher in TBI than in controls, regardless of the PMI (PMI tested 0–12 h). Lactic acid was significantly higher in TBI than controls only at the time of death and 12 h after death; hypoxanthine was significantly higher in TBI than controls at 6 and 12 h after death.
			Serum	TNFα and IL-1β were significantly higher in TBI than in controls, regardless of the PMI (PMI tested 0–12 h). HMGB1 was significantly higher in TBI than in controls at 6 and 12 h after death.
Kobeissy et al. 2022 [22]	Original research	Animal	Serum	IL1- β, IL-6, and IL-10 were significantly higher in TBI than in controls.
**Tissues**				
**References**	**Type of Paper**	**Model**	**Tissue**	**Brief Description**
Njau et al. 1991 [23]	Original research	Animal	Skin	In wounded skin, Mg^2+^ decreased within 30 min, increased and peaked at the 2nd hour after wounding, then gradually decreased until the 8th hour. Ca^2+^ increased within 1 h after wounding, then decreased; however, at the 4th hour, it increased again until the 8th hour. Zn^2+^ increased within the first 120 min, then decreased gradually until the 8th hour. No statistical significance was found among different sites of sampling (the lesion, 2 cm from the lesion, and 4 cm from the lesion). Survival time tested in this study: 30 s; 30 min; 1, 2, 4, and 8 h after wounding.
Chen et al. 1995 [24]	Original research	Human	Skin, muscle	Fe^2+^ concentration in vital wounded skin (different types of lesions) was significantly higher than in controls (not injured skin of the same subjects). Survival time ranged from 5 min to 6 h. PMI ranged from 24 to 72 h.
He et al. 1996 [25]	Original research	Human	Skin	LTB4 was only detectable in vital skin lesions and not in wounds inflicted after death. It was also detectable in formalin-fixed injured skin if fixation < 10 days. PMI ranged from 4 h to 1 day.
Laiho et al. 1998 [26]	Original research	Animal	Skin	MPO was high in vital skin lesions, but no comparison with normal skin or PM injured skin controls was done. MPO activity depended on blood loss (decreased activity with 35% loss of blood), the depth of the lesion (deeper lesions, higher activity), and skin thickness (thicker skin, higher activity).
Grellner et al. 2000 [27]	Original research	Human	Skin	The study found great interindividual variability in cytokine levels. In autoptic samples, IL-1β levels were significantly higher in wounded skin than in controls (normal skin) only when the wound age was ≤5 min. IL-6 levels were significantly higher in wounded skin than in controls when the wound age was ≤5 min and >24 h. TNF-α levels were significantly higher in wounded skin than in controls only when the wound age was ≤5 min.
Laiho et al. 2004 [28]	Original research	Animal	Skin	Significantly increased albumin levels in vital skin lesions (incision, excoriation, heat, and freezing injuries) with different ages (from 5 min to 15 days for incision, 5 min to 4 weeks for excoriations, and 60 min to 2 weeks for heat and freezing injuries) and sampled soon after death. Still significantly increased in incision wounds aged 15 and 30 min and excoriation aged 30 and 60 min when sampled 3 days after death.
Barnes et al. 2009 [29] *	Original research	Animal	Skeletal muscle	MT1-MMP levels significantly decreased in muscular injuries aged 48- and 72-h post-injury, compared to controls. TIMP-2 protein was decreased muscular injuries aged 3- and 48-h post-injury, compared to controls.
Kagawa et al. 2009 [30]	Original research	Animal	Skin	In vital lesions, C-fos, fosB, and MKP-1 peaked 1 h after injury; CD14 and CCL9 peaked between 12 and 24 h after injury, while PlGF and MCP-5 before 5 days after injury.
Takamiya et al. 2009 [31]	Original research	Human	Skin	IL12+ in less than 30 min after injury, at 2 h IL5, IL13, and IL17+. By 9 h after wounding MCP1, IL1ß, G-CSF, and MIP1ß showed+, while IL5 and IL13 peaked. IL17+ increased until 33–49 h and IL 12+ until 71–116 h. IL7 negativity from early phases of wound healing.
Gao et al. 2012 [32]	Original research	Animal	Brain	HMGB1 decreased in the first 6 h after TBI, coming back to baseline in 2 days. RAGE increased 1 h after TBI and peaked 6 h after TBI. PMI tested 6–72 h.
Fan et al. 2014 [33] *	Original research	Animal	Skeletal muscle	In vital skeletal muscle injuries, α7nAChR and GAPDH levels significantly increased from 12 h to 14 days post-wounding.
Yang et al. 2014 [34]	Original research	Animal	Brain	RT-PCR was used to detect β-APP mRNA. β-APP concentrantion increased between 1 and 6 h in PBSI.
Kimura et al. 2015 [35]	Original research	Animal and human	Skin	LC3-II levels decreased, while p62 levels increased in mice with vital wounds when the survival time was longer than 30 min; the PMI did not influence these findings (PMI ranged between 1–4 days). In autoptic samples, LC3-II levels were reduced, while p62 levels increased in controls (PMI tested < 24 h).
Birincioğlu et al. 2016 [36]	Original research	Human	Skin	TNF-α was higher in wounded skin than in controls in all age wounds, except in 2–4 h-old lesions. IL-6 was higher in wounded skin than in controls but had statistical significance only when the wound ages were <30 min and >18 h. No statistical significance for other cytokines.
Tian et al. 2016 [37] *	Original research	Animal	Skeletal muscle	PAX7 levels increased 1-day post-injury, and the highest level was found at 5 days. MyoD increased after 1 day after the lesion.
Wang et al. 2016 [38]	Original research	Animal	Skin	In vital lesions, MCP-1 and CXCL12 increased between 12 h and 5 days after injury. IL-1 β, TNF-α, and pro-col IIIa1 increased 7 days after injury. IL-6 and VEGF-A raised 12 h until 10 days after injury. Procol Ia2 increased from 7 days to 21 days, while IFN decreased between 12 h and 10 days after injury.
Peréz et al. 2017 [39] *	Original research	Human	Skin	Fe^2+^ and Zn^2+^ concentrations were higher in injured skin than in controls. PMI tested for 19–36 h.
He et al. 2018 [40] *	Original research	Animal and human	Skin	No statistical differences in CXCL1 and CXCR2 concentrations between vital lesions and control have been found.
Qu et al. 2019 [41] *	Original research	Animal and human	Skin	No statistical differences in ATF3 concentration between vital lesions and control have been found.
Peyron et al. 2021 [42]	Original research	Human	Skin	In vital injuries, IL-1β, IL-4, IL-6, IL-10, IL-12p70, IL-13, and TNF-α levels were higher than controls. PMI tested 66.3 +/− 28.3 h.

**Table 2:** The results of our review on immunohistochemical studies on wound vitality (84 articles). * These studies included different kind of analysis to investigate the differential protein expression in tissues, and they have been included in both the relative tables. + indicates positive/positivity; α7nAChR, α7 nicotine acetylcholine receptor; αlact, αl antichymotrypsin; α2m, α2 macroglobulin; A1-ACT, alpha1-antichymotrypsin; RM3/1, anti-CD163 marker; 25F9, mature macrophages marker; AM, ante-mortem; DC-SIGN, dendritic cell-specific intercellular adhesion molecule-3-grabbing non-integrin; GM-CSF, granulocyte macrophage-colony stimulating factor; GPA, glycophorin A; HSP, heat-shock protein; ICAM-1, intercellular adhesion molecule 1; IHC, immunohistochemistry; IL, interleukin; iNOS, inducible nitric oxide synthase; mAb, monoclonal antibody; MCP, monocyte chemoattractant protein; MHC-II, major histocompatibility complex II; MMP, matrix metallopeptidase; PM, post-mortem; PMI, post-mortem interval; PMNs, polymorphonuclear cells; PTI, post traumatic interval; RAGE, receptor for advanced glycation endproducts; TNF-α, tumor necrosis factor α; VEGF, vascular endothelial growth factor; VCAM-1, vascular cell adhesion molecule-1; ATP, adenosine triphosphate; TN, tenascin; FN, fibronectin; MRP, myeloid-related protein; FGF, fibroblast growth factor; M-CSF, macrophage colony-stimulating factor; MIG, monokine inducible by interferon gamma; PDGF, platelet-derived growth factor; ORP, oxygen regulated protein; HLA, human leukocyte antigen; TGF, transforming growth factor; MPO, myeloperoxidases; COX, cyclooxygenase; pAb, polyclonal antibody; CB2R, cannabinoid receptor type 2; MNC, mononuclear cell; FBC, fibroblastic cell; SP, surfactant protein; HIF, hypoxia inducible factor; AQ, aquaporin; Cath, cathepsin; MIP, macrophage inflammatory protein; CML, carboxymethyllysine; Flk, receptor for vascular growth factor; EPC, endothelial progenitor cell; TIMP, metallopeptidase inhibitor; Chil, chitinase-like; SMA, smooth muscle actin; FLIP, FLICE inhibitor protein; INF, interferon; Ub, ubiquitin.

References	Type of Paper	Model	Tissue	Brief Description
Betz et al. 1992 [43]	Original research	Human	Skin	Fibronectin + in the skin from a few seconds to 6 weeks after wounding (with a different distribution pattern than control cases).
Betz et al. 1993 [44]	Original research	Human	Skin	Fibronectin + in skin lesions from a few minutes after wounding. A1-ACT provides no information.
Fieguth et al. 1994 [45]	Original research	Human	Skin	αlact, α2m, and lysozyme showed false + reaction in post-mortem wounds.
Betz et al. 1995 [46]	Original research	Human	Skin	Macrophage maturation markers were evaluated. RM 3/1+ cells increase 7 days after wounding, as well as in 7-month-old scar tissue. 25F9+ cells increase 11 days after wounding, as well as in 3-month-old scar tissue.
Kondo et al. 1996 [47]	Original research	Animal	Skin	IL-α, IL-ß, IL-6, and TNFα + in 3–6 h-old wounds in neutrophils; by 24 h, they were then substituted by macrophages. TNFα and IL-ß levels increased soon after wounding and peaked at 3 h. IL-α showed a peak at 6 h after wounding, while IL-6 peaked at 12 h after injuring.
Dressler et al. 1997 and Dressler et al. 2000 [48,49]	Original research	Human	Skin	ICAM-1 (CD54) strongly + in vital wounded skin, while only slightly + in undamaged skin. The earliest + was at 15 min after wounding, strong + 4 h after wounding, with still + reaction even 10 days after wounding. The distribution of ICAM-1 expression was different between autopsy (PM sampling) and surgical (AM sampling) cases. In autopsy cases, the PMI was ≤7 days.
Fieguth et al. 1997 [50]	Original research	Human	Skin	The study aimed to evaluate the influence of post-mortem clamping and autolysis on the IHC reactions by testing antibodies against αlact, α2m, fibronectin, and lysozyme. Autolysis produced an increase in false + reactions, while post-mortem clamping did not.
Dressler et al. 1998 [51]	Original research	Human	Skin	P-selectin + from 3 min up to 7 h after wounding, different distribution pattern between autopsy (PM sampling) and surgical (AM sampling) cases. E-selectin + from 1 h up to 17 days after wounding, with a decrease after the 12th hour. L-selectin was not significant.
Grellner et al. 1998 [52]	Original research	Animal	Skin	Fibronectin moderately + in wounded areas, whereas in normal skin, both the epidermis and blood vessels showed strong +.
Tabata 1998 [53]	Original research	Human	Skeletal muscle	Fibronectin + in muscles when the death occurred immediately after injury and within 2 h after injury. GPA + indicated bleeding in cases of putrefactive changes. Myoglobin (Mb) − reaction in opaque fibers.
Dressler et al. 1999 and Dressler et al. 2000 [54,55]	Original research	Human	Skin	VCAM-1 (CD106) strongly + in vital wounded skin, while only slightly + in undamaged skin. Strong + was present from 3 h up to 3.5 days after wounding, with a decrease after the 4th-6th hours.
Kondo et al. 1999 [56]	Original research	Human	Skin	At 4 h after wounding, IL-α + neutrophils remained + until 24 h. Neutrophils were substituted by macrophages and fibroblasts, both IL-α +, as skin repair progressed.
Grellner et al. 2000 [27]	Original research	Human	Skin	IL-1ß decreased reactivity in surgical samples by 30 min but increased at 2 h after wounding; in autopsy samples, IL-1ß strongly + by 24 h after injury. IL6 strongly + in autopsy samples from 1 h to at least 24 h after injury. TNF-α was strongly + in surgical samples at 1 h after surgery, while in autopsy samples, its levels were higher, between 1 to 24 h.
Kondo et al. 2000 [57]	Original research	Human	Skin	c-Fos and c-Jun weak + in neutrophils’ nuclei at 24 h after wounding. In later phases, c-Fos and c-Jun + reactions in macrophages and fibroblasts in granulation tissue.
Psaroudakis et al. 2001 [58]	Original research	Animal	Skin	Alkaline phosphatase + at 3.5 h after wounding and peaked at 32 h, nonspecific esterases + at 1 h after wounding and peaked at 24 h and ATPase + at 2 h after wounding and peaked at 20 h.
Grellner 2002 [10]	Original research	Human	Skin	IL-1ß + by 15 min, increasing levels were shown at 30–60 min old wounds and remained stable until 8 days postmortem. IL6 + by 20 min after injury; stronger + by 60–90 min until 5 h. TNF-α + reaction after 15 min, strongly + at 60–90 min after wounding.
Hausmann et al. 2002 [59]	Original research	Human	Brain	MIB-1 + in cortical contusions in 3 days old wounds and showed an increasing trend until 14 days. Weak + could still be detected 4 weeks post-trauma.
Kondo et al. 2002 [60]	Original research	Human	Skin	IL-8, MCP-1, and MIP-1α + neutrophils from 4 to 12 h. IL-8, MCP-1, and MIP-1α cytoplasmic + in macrophages and fibroblasts, granulation tissue formation, and angiogenesis tissue.
Kondo et al. 2002 [61]	Original research	Animal and Human	Skin	Animal samples: Ub strongly + neutrophils at 12 h post wounding; between 12 h and 6 days, decreasing levels of neutrophils, while macrophages increased; at day 6, Ub + fibroblasts and macrophages. Human samples: from 4 h to 24 h, Ub + neutrophils cells at the wound site; with increasing wound age, infiltration of macrophages, then fibroblasts.
Ortiz-rey et al. 2002 [62]	Original research	Human	Skin and muscle	TN + in the basement membrane of blood vessels and skin appendages, slightly + reaction in the papillary dermis. FN + at the wound edge and adjacent dermis, the prevalence of reticular pattern in vital wounds, rather than in postmortem cases. FN and TN strongly + in hemorrhages both in vital and postmortem samples.
Ortiz-rey et al. 2002 [63]	Original research	Human	Skin and muscle	In situ end-labelling technique (Apop-Tag) in 30 human sugical skin injuries with age since injury ranging from 3 min to 8 h and found that apoptotic keratinocytes are found in over 50% of the cases with a post-infliction interval of at least 120 min
Bonelli et al. 2003 [64]	Original research	Human	Skin	Tryptase + and chymase + cell (mast cells) densities were higher in vital skin lesions than controls in a time interval < 5 min up to 60 min after wounding.
Fieguth et al. 2003 [65]	Original research	Human	Soft tissue	Myoglobin depletion in muscle fibers. Fibronectin strongly + in lymph and blood vessels, as well as damaged skeletal muscle, and areas of hemorrhage. C5b-9 intense staining in sarcolemma fibers and intracellular areas. MRP14 strongly + in perivasal inflammatory infiltrates.
Fieguth et al. 2003 [66]	Original research	Human	Skin	Fibronectin was minimally detected in wounds that occurred during immediate fatal trauma, while highly + in areas of active bleeding in immediate fatal wounds. Fibronectin strongly + at 20 min after injury; by 40 min, massive + could be detected. MRP8, MRP14, and defensin + reactions at 20–30 min after wounds occurred could still be demonstrated in 2 to 30 days old wounds.
Gauchotte et al. 2003 [67]	Original research	Human	Skin	FVIIIra strongly + in autopsy and surgical samples; putrified samples stained strongly for FVIIIra. CD15 strongly + at wound margins in both autopsy and surgical samples, at a minimum of 9 min after injury. After 7, 14, and 21 days of putrefaction, sensitivity values decreased remarkably. Tryptase + in autopsy and surgical samples.
Ortiz-rey et al. 2003 [68]	Original research	Animal	Skin and muscle	FN and TN strongly + reticular staining in vital skin samples from 5 min after wounding to 15 min. Slightly + reaction in postmortem samples. FN and TN weak + in intracellular muscle fibers, but not statistically significant.
Hayashi et al. 2004 [69]	Original research	Human	Skin	VEGF negative reaction until 24 h after wounding, with increasing wound age VEGF + cytoplasm of mononuclear cells and fibroblastic cells, around CD31 + neovessels.
Balažic et al. 2005 [70]	Original research	Human	Skin	Fibronectin + in head gunshot skin in cases of a longer survival time, weaker + when the survival time was extremely short.
Bacci et al. 2006 [71]	Original research	Human	Skin	TNF-α + (mast cells) from 15 min after wounding was significatively more intensive than controls.
Tarran et al. 2006 [72]	Original research	Human	Skin	In post-mortem samples, neutrophil elastase + from 12 h to 7 days, CD68 + from day 2 to day 28, and CD45 + (lymphocytes) from day 35 to day 77.
Takamiya et al. 2007 [73]	Original research	Human	Skin	Neutrophil elastase and CD68 + (macrophages) from 2 h post-injury, peaking at 33–49 h, while CD3 + (lymphocytes B) from 71 h and vimentin + (fibroblasts) from 246 h. TNF-α+ from 30 min, peaked at 9 h after wounding. IL10, GM-CSF, and IFN-γ + from 2 h after injury, with peaks, respectively, at 71–116 h, 33–49 h, and 12–15 h. IL6, IL8, and IL2 + peak at 9 h. IL4+ peak at 33–49 h.
Takamiya et al. 2007 [74]	Original research	Animal	Brain	27 different cytokines expression in different phases of cerebral wound healing. IL12 p40, IL18, bFGF, KC, M-CSF, MIG, MIP-1α, and PDGF BB were strongly expressed after cerebral stab wounds in mice.
Ishida et al. 2008 [75]	Original research	Human	Skin	ORP150 + mononuclear and fibroblastic cells at 24 h after wounding. ORP150 + non-enhanced in PM inflicted wounds.
Ortiz-Rey et al. 2008 [76]	Short communication	Human	Skin	CD31 + in endothelial cells, P-selectin + reaction in small blood vessels adjacent to the vital wound edge. P-selectin and CD31 weakly + in PM wounds and presence of background artifact or tissue disruption.
Neri et al. 2009 [77]	Original research	Human (liveborn and stillborn fetuses)	Umbilical cords	Tryptase (mast cells), α1-antichymotrypsin, and CD68 were strongly + in umbilical cords of liveborn fetuses, while weakly reactive in stillborn. Among stillborn fetuses, CD68 + was higher in perinatal deaths during prolonged labor than intrauterine deaths.
Nogami et al. 2009 [78]	Original research	Animal	Skin	Podoplanin + vessels absence at 1, 3, 7, 28, 56, and 84 days after incision. From day 5 to 7, CD31+ vascular vessels with a mainly vertical course; after 14 days, CD31+ vascular vessels result to be less vertical; by day 28, became similar to vessels in control skin areas. In paraffin sections, vWF + lymphatic vessels in wound areas and absence of podoplanin + vascular vessels.
Oehmichen et al. 2009 [79]	Original research	Human	Brain	CD68 + macrophages in cortical hemorrhages at 3 h PTI, CD68 weak + in the adjacent cortex at 12 h PTI, strongly + by 60 h PTI, peak at day 10 PTI. HLA-D + macrophages at 6 h PTI in hemorrhages, peak at day 12. HAM-56 reactive macrophages within 31 h PTI, increasing up to day 11. LN-5 + macrophages in hemorrhagic areas detect at 24 h PTI, strongly + on day 5. 25F9 slightly + macrophages within 100 h PTI
Bohnert et al. 2010 [80]	Original research	Human	Lung	Fibronectin + in the lung tissue of burned corpses (intravital fire exposure) was more intensive than controls.
Cattaneo et al. 2010 [81]	Original research	Human	Bone	GPA + in vital bone fractures, survival time ranged from 34 min to 26 days.
Jin et al. 2010 [82]	Original research	Animal	Oral mucosa	TNF-α strongly + on day 3 after surgery, decreasing by day 5–7, TNF-α+ neutrophils on day 1 to 3, and fibroblasts on day 14 post-surgery. TGF-ß1 levels decreased on day 5 after surgery and then increased until day 14.
Guler et al. 2011 [83]	Original research	Human	Skin	Tenascin strongly + in all types of wounds investigated, by 24 h after injury. Weak + of ubiquitin since 24 h after wounding, still present in wounds over 40 days old, while tenascin was negative.
Ryu et al. 2011 [84]	Original research	Animal	Oral mucosa	TNF-α+ 1-day post-surgery in scalpel wounds and 3 days post-surgery in laser wounds, reaching its peak at day 3 for all groups of surgery. TNF-ß + levels increased 3 days post-surgery, decreased until day 7, and increased further until day 14 both in laser and scalpel wounds. The highest intensity is shown at day 3.
Taborelli et al. 2011 [85]	Original research	Human	Skin	GPA+ at day 3, 6, and 15 PM, negativity after day 30, in putrefied specimens in air, while histological techniques were no longer useful by day 15. GPA + on day 3 and day 6 in putrefied specimens in water, while histological techniques were no longer useful by day 6.
Capatina et al. 2012 [86]	Case report	Human	Skin and liver	Fibronectin + in the skin and liver samples, suggesting vital lesions. P-selectin and tenascin-X were negative/irrelevant.
Cecchi et al. 2012 [87]	Case report	Human	Skin	P-selectin + in wounded skin, while E-selectin was negative. The authors concluded the survival time was less than 30–60 min.
Ishida et al. 2012 [88]	Original research	Human	Skin	MPO, and COX2 + neutrophils at wound sites from 2 h to 2 days after injury. In 3-day-old specimens, CD68 + macrophages were present and reactive for COX2 and pAbs.
Zheng et al. 2012 [89]	Original research	Animal	Skin	CB2R + from 1 to 12 h in PMNs on days 1 and 3 in round-shaped MNCs, as well as in FBCs from day 5. Decreased + from day 14 in MNCs, and day 21 in FBCs.
Capatina et al. 2013 [90]	Original research	Human	Skin	Fibronectin and P-selectin + in skin lesions with a short survival time. The PMI did not influence their expression.
Agha et al. 2013 [91]	Original research	Animal	Skin	iNOS and VEGF + in wounded skin. iNOS started after 6 h, peaked on day 1, still + on day 10 after wounding. Weak iNOS + also in normal skin. VEGF started from day 1, strong + from day 3 to 10 after wounding.
Akbaba et al. 2014 [92]	Original research	Animal	Skin	Ki-67 + in the skin from day 1 to day 5 after wounding. Ubiquitin + from >24 h up to 7 days after wounding.
Bacci et al. 2014 [93]	Original research	Human	Skin	MHC-II+ and CD1a+ cells increase in the epidermis after wounding, the MHC-II+/CD1a+ cells ratio was lower than controls within 30 min, then higher from 30 min up to 24 h after wounding. In the dermis, MHC-II+ cells increase between 31 and 60 min after wounding, while DC-SIGN+ and CD11c+ cells were seen at the periphery of infiltrates and in the basal epidermal layer.
Cecchi et al. 2014 [94]	Original research	Human	Lung	SP-A massive + in intra-alveolar deposits in cases of intense hypoxic stimulus. HIF1-α + in vessels in cases of hypoxia, the intensity was proportional to the duration of the hypoxic stimulus.
Fan et al. 2014 [33] *	Original research	Animal	Skeletal muscle	Slight α7nAChR + in sarcolemma and sarcoplasm of undamaged myofibers. Presence of α7nAChR + PMNs, macrophages, and myofibroblasts in contused skin, starting from 1–3 h after contusion (only a few cells), increasing from the 12th hour, peaking at day 7, and gradually reducing within the 14th day after wounding.
Kubo et al. 2014 [95]	Original research	Animal and Human PM	Skin	AQ3 + keratinocytes in the area surrounding burned skin, similarly to control samples. No difference in the expression of AQ3 between ante- and post-mortem burned skins.
Montisci et al. 2014 [96]	Original research	Human	Skin	Cath-D + in both surgical and PM samples is homogeneous in different sampling timing. From 30 min after injury, Cath-D is strongly + in surgical samples.
Van de Goot et al. 2014 [97]	Original research	Human	Skin	Fibronectin, CD62, and Factor VIII strongly + in wounds that occurred a few minutes before death, these markers + increased in 30-min-old wounds.
Balandiz et al. 2015 [4]	Original research	Animal	Skin	IL-1β+ cells in the epidermis from 2 h (peak) up to 72 h from hanging (putrefactive phenomena independent).
Capatina et al. 2015 [98]	Case report	Human	Skin	Fibronectin and P-selectin + reaction in skin wounded 1 h after death (only post-mortem lesions).
El Deeb et Badr El Dine 2015 [99]	Original research	Animal	Skin	mAb D2–40 (lymphatic endothelium marker, it reacts with M2A antigen) + in the peripheral granulation tissue, edge, and deep of the wound. Positivity ranged from day 3 to day 7 in sutured wounds, from day 5 to day 10 in wounds that were not sutured.
Fronczek et al. 2015 [100]	Original research	Human	Skin	Neutrophilic granulocytes peaked at 0.2 to 2 days after injury and declined gradually in time. CD45 + lymphocytes peaked at 0.2 to 2 days and declined by day 10 after wounding. CD68 + macrophages peaked at days 2 to 4 after injury and declined gradually in time. MIP-1, and IL8 + from 0.2 to 2 days old wounds; NεCML strongly + at 0.2 to 2 days old wounds, decreasing by day 4 to 10, then increasing in wounds older than 10 days.
Ishida et al. 2015 [101]	Original research	Animal	Skin	CD34 and Flk-1 + EPCs cells accumulate at wound sites, while scarcely detected in unwounded skin tissue samples
Kara et al. 2016 [102]	Original research	Animal	Skin	Collagen I and Collagen III + fibroblasts, and VEGF + inflammatory cells, at 3 h after wounding, decreasing by 6 and 24 h. E-selectin and fibronectin + fibroblasts, strongly reactive by 1 h after injuring until 24 h. IL-α+ fibroblasts were statistically significant at 3 h and maintained until 24 h. P-selectin and TGF-ß1 + inflammatory cells in the 1-h-old wound.
Yu et al. 2016 [103]	Technical note	Animal	Skeletal muscle	MMP-2 and TIMP-2 + in PMNs from 6 to 24 h after injury in the skeletal muscle, and MNCs in the contused zones. From day 3, MMP-2 intense + in centronucleated myotubes. MMP-2 and TIMP-2 + were also found in endothelial cells of new vessels.
Abo El-Noor et al. 2017 [104]	Original research	Animal	Skin	iNOS + in burned skin. Starting from day 1, peak at day 7, declining from day 9.
Ji et al. 2017 [105]	Original research	Animal	Skin	RAGE + PMNs from 6 to 12 h after wounding, RAGE + MNCs from day 1 to 3 post-injury, and day 5 post-wounding RAGE reactivity primarily in MNCs and fibroblasts.
Legaz et al. 2017 [106]	Original research	Human	Skin	Cathepsin D moderative or strongly + cells in skin wounds of ligature marks. Cathepsin D and P-selectin moderately + cells in skin wounds with subcutaneous injury, rather than subcutaneous and muscular injury.
Murase et al. 2017 [107]	Original research	Animal	Skin	Chil3 + cells from day 1 to day 9 after wounding. From day 2, presence of two types of + cells, a small oval one and large elongated one.
Doberentz et Madea 2018 [108]	Case series	Human	Heart, lung, and kidney	Heart, lung, and kidney samples were HSP27 and HSP70 negative in two burned-after-death corpses. Renal tissue was moderately + for HSP27 reaction, while negative for HSP70, in a case of death with immediate burning (bomb explosion). The authors considered the HSP27 expression a supravital phenomenon.
Ishida et al. 2018 [109]	Original research	Human	Skin	AQ1 weakly + at day 2 after injury, by day 3 to 14 was detected a stronger + to AQ1. AQ3 weakly + in wounds from 3 to 14 days old.
Ishida et al. 2018 [8]	Original research	Human	Skin	AQ1 + in dermal capillaries of ligature marks, while AQ3 was primarily expressed in keratinocytes of ligature marks.
Legaz Pérez et al. 2018 [39] *	Original research	Human	Skin	Fibronectin + in basement membranes and interstitial connective tissue of ligature wounds. Cathepsin D + in skin wounds of hanging marks while P-selectin showed weaker + reaction in vital wounds compared to normal skin.
Metwally et al. 2018 [110]	Original research	Human	Skin	Decreasing intensity of both P-selectin (CD62p) and fibronectin as the PM interval increases until 12 h. CD62p and fibronectin increased intensity from 30 min to 90 min after injury in AM samples.
De Matteis et al. 2019 [111]	Original research	Human	Skeletal muscle	Intracytoplasmic depletion of troponin I in neck muscle fibers in cases of suicidal hanging.
Focardi et al. 2019 [112]	Original research	Human	Skin	MHC-II + dendritic cells were significantly higher in ligature marks and vital lesions. CD1a + Langerhans cells were higher in vital lesions and ligature marks, as well.
Khalaf et al. 2019 [113]	Original research	Animal	Skin	α-SMA and VEGF negative to mild expression at 0, 1, and 3 days after wounding. CD68 + macrophages from day 3 at the wound surface, peaking at day 7, and declining by day 14. α-SMA was still strongly expressed by day 14.
Focardi et al. 2020 [114]	Original research	Human	Skin	iNOS + Langherans cells were higher in ligature marks than other samples groups. iNOS + mast cells in vital wounds and hanging furrows. MHC + mast cells were highly expressed in the sulcus, while barely visible in vital wounds, controls, or post-mortem wounds.
Maiese et al. 2020 [115]	Original research	Human	Skin	Intracytoplasmic depletion of FLIP in epidermal layers, with epidermal flattening, in subjects who died by hanging.
Baldari et al. 2021 [116]	Original research	Human	Bone and soft tissues	GPA + in vital bone fracture and wounded soft tissues in corpses at different putrefactive stages (PMI range 2–187 days).
Bertozzi et al. 2021 [117]	Original research	Human	Skin and soft tissues	Tryptase, GPA, IL15, CD15, CD45, and MMP9 + in vital wounded putrefied skin (PMI < 15 days) with a differential time expression, according to the PMI.
Niedecker et al. 2021 [118]	Original research	Animal and human	Muscle and myocardium	Human skeletal muscle stained MMP-9 and MMP-2 + from a few minutes after the injury to 12 h, and TIMP-1 + from a few minutes to 4 h. MMP-9, TIMP-1, and MMP-2 were strongly + in human myocardium injuries from a few minutes to 4 h. TIMP-1 negativity in rats’ heart postmortem inflicted wounds.
Peyron et al. 2021 [42] *	Original research	Human	Skin	IL-8 + cells in five vital skin wounds, while no IL-8 + cells in the controls.
Prangenberg et al. 2021 [119]	Original research	Human	Skin	AQ3 strongly + in injured epidermis, independent of kind of injury, while slightly + in uninjured skin.
Wegner et al. 2021 [120]	Case report	Human	Kidney, lung, and skin	The study described only two cases. In the first case, the body was found in a sauna after 3 days. AQ3 + in the epidermis, HSP 27, HSP 60, and HSP70 were not detectable in kidneys or lungs. In the second case, the body was found in a sauna after about 35 min, with HSP 27, HSP 60, and HSP 70 + in preserved lung and kidney tissue.
Khismatullin et al. 2019 [121]	Original research	Human	Blood clot	Picro-Mallory staining determines different fibrin color depending on clots formation timing. At 30 minutes to 6 h of maturation, fibrin stained red, from 6 to 12 h fibrin was purple or violet while in old clots, incubated 24 to 48 h, fibrin stained blue.
Zamboni et al. 2004 [122]	Original research	Human	Skin	FXIII positive effects against MMPs action on fibroblasts cultures, enhancing wound healing.

**Table 3:** The results of our review on mRNAs and miRNAs quantified for wound vitality evaluation (respectively, 20 and 21 papers). * α7nAChR indicates α7 nicotine acetylcholine receptor; IL, interleukin; MMP9, matrix metallopeptidase; TNF-α, tumor necrosis factor α; TIMP-2, tissue inhibitor of metalloproteinases; MCP-1, monocyte chemoattractant protein 1; MT1-MMP, membrane type-1 matrix metalloproteinases; COX, cyclooxygenase; SNAT2, amino acid transporter2; CMA, chymase; CCL, C-C motif chemokine ligand; Fosl1, FOS-like 1; MyOD, myoblast determination protein; FDZ4, frizzled-class receptor 4; SFRP5, secreted frizzled-related protein 5; CSF, colony stimulating factor; PAI1, plasminogen activator inhibitor 1; Pax7, paired-box protein 7; TGF; transforming growth factor; TNNI2, troponin I 2; FGF, fibroblast growth factor; TNMD, tenomodulin; FOXC, forkhead box C; PROX, prospero homebox; TBI, traumatic brain injury; VEGF, vascular endothelial growth factor; CXCL, C-X-C motif chemokine ligand; CXCR, C-X-C motif chemokine receptor; DUSP, dual specificity phosphatase; AFT, activating transcription factor; KCNJ, potassium inwardly rectifying channel subfamily J; EMT, epithelial to mesenchymal transition.

**Messenger RNA**				
**References**	**Type of Paper**	**Model**	**Tissue**	**Brief Description**
Ohshima et al. 1998 [121]	Original research	Animal	Skin	IL-10 mRNA increased at 15 min after injury, peaked at 60 min, and maintained an increase until 5 days.
Sato et al. 2000 [122]	Original research	Animal	Skin	IL-6 mRNA peaked at 6 h after injury. IL-1α, IL-1β, and TNFα peaked between 48 and 72 h after injury.
Iino et al. 2003 [123]	Original research	Animal	Brain	FE65 mRNA was significantly increased in TBI rats’ brain tissue than in controls when the survival time was 30 min and 1 h; no statistical significance when the survival time was >1–24 h. FE65 mRNA peaked in 1 h and decreased 12 h post-injury, another peak was documented at 24 h post-injury, coming back to baseline at 48 h post-injury.
Takamiya et al. 2003 [124]	Original research	Animal	Brain, skin, kidney, and liver	In skin injuries, bFGF mRNA peaked 1 h post-injury. In hepatic injuries, it increased after 24 post-injuries. In cerebral injuries, it increased within 1 h and peaked at 48 h post-injuries. In renal injuries, it increased within 24 h post-injuries.
Bai et al. 2008 [125]	Original research	Animal	Skin	IL-1 β mRNA increased within 30 min post-injury, peaked 2 h, and decreased 2 days after injury. COX-2 mRNA increased at 1 h post-injury, peaked at 3 h, and came back to baseline in 3 days. MCP-1 mRNA increased 3 h post-injury, peaked at 5 h post-injury, and came back in 7 days.
Barnes et al. 2009 [29] *	Original research	Animal	Skeletal muscle	MT1-MMP 1 mRNA decreased 24–72 h after injury; MT1-MMP 2 and TIMP-2 mRNA increased within 10–72 h after injury, and MT1-MMP 9 mRNA increased in 10–48 h after injury.
Sun et al. 2010 [126]	Original research	Animal	Skin	In vital contusions, troponin I mRNA decreased at 30 min, 1h, and 6 h after injury, compared to controls.
Sun et al. 2012 [127]	Original research	Animals	Skeletal muscle	In vital injuries, all mRNAs are still evaluable at 6 and 12 h after injury.
Du et al. 2013 [128]	Original research	Animal	Skeletal muscle	SNAT2 mRNA increased at 4, 16, 20, and 24 h after injury, compared to controls or postmortem injuries.
Fan et al. 2014 [33] *	Original research	Animal	Skin	α7nAChR mRNA increased to 2.65-fold at 7 days post-injuries.
Palagummi et al. 2014 [129]	Original research	Human	Skin	DUSP1 mRNA, IL7 mRNA, TNFα mRNA, and VEGFA mRNA increased until 6 days post- injury. IL1β mRNA increased within 12 h post- injury, and CMA1 mRNA increased 10 min post-injury.
Kameyama et al. 2015 [130]	Original research	Animal	Skin	CXCL2, CSF3, MMP9, PAI1, and CSF2 increased within 3 days post-injury, while at the same time, TGFα, TNNI2, FGF1, TNMD, leptin, and CXCL12 mRNA FOXC2, PROX1, and FGF2 mRNA showed a significant decrease.
Wang et al. 2016 [38] *	Original research	Animal	Skin	MMP-2 mRNA increased between 1 and 7 days, peaked on the 7th day, and decreased between 10 and 14 days after injury. IL6 and CCL-2 mRNA peaked at 1 day. MMP-9, TNF-α, and CCL3 mRNA peaked at 5 days after injury. CCL5 maintained persistently increased and reached the baseline on the 5th day after injury.
Yu et al. 2016 [103] *	Original research	Animal	Skeletal muscle	MMP-2 mRNA increased at 3 days after injury, while TIMP-2 mRNA increased at 5 days after injury.
Tian et al. 2016 [37] *	Original research	Animal	Skeletal muscle	MyoD mRNA increased after 1 day post-injury, peaked at 3 days, maintained increased until 7 days post-injury. Pax7 mRNA peaked between 3–7 days post-injury.
Zhu et al. 2016 [131]	Original research	Animal	Skin	In vital injuries, FZD4 mRNA increased within 12 h post-injury, decreased between 12- and 28-h post-injury, while a second peak was described 40 h after injury. SFRP5 mRNA decreased in wounded samples, the lowest levels were found at 20 h after injury. Fosl1 mRNA levels are strongly overexpressed the first 24 h after injury, then slowly decreased until 48 h.
He et al. 2018 [40] *	Original research	Animal and human	Skin	In rats in vital injury, CXCL1 mRNA increased until 96 h after injury; CXCR2 mRNA increased until 72 h after injury. In humans, CXCL1 mRNA and CXCR2 mRNA increased until 48 h after autopsy.
Ye et al. 2018 [132]	Original research	Animal and human	Skin	IL-6 and IL-20 mRNA levels in mice are increased in vital injuries (inflicted 30 min before death) and remained elevated until 72 h after death occurred. In human samples, IL-6 and IL-20 levels increased in wounded skin.
Qu et al. 2019 [41] *	Original research	Animal and human	Skin	AFT3 mRNA upregulation until 48 h after autopsy in wounded samples. In rats, ATF3 mRNA increased until 96 h after death, while BTG2 mRNA increased until 48 h after death.
Du et al. 2020 [133]	Original research	Animal	Skeletal muscle	In vital injuries, all mRNAs are detectable between 4 and 48 h post-injury.
**MicroRNAs**				
**References**	**Type of Paper**	**Model**	**Tissue**	**Brief Description**
Yu et al. 2010 [134]	Short communication	In vitro	Skin	miR-205 and miR-184 upregulation in epithelial cells after scratch wound regulate keratinocyte survival signaling.
Bertero et al. 2011 [135]	Original research	In vitro and animal	Skin	miR-483–3p upregulation in scratch wound in human cultures and wounded skin in mice.
Yang et al. 2011 [136]	Short communication	Animal	Skin	miR-21 upregulation improve healing in excisional wounds by promoting keratinocytes migration and re-epithelialization.
Pastar et al. 2012 [137]	Original research	In vitro and animal	Skin	miR-16, miR-20a, miR-21, miR-106a, miR-130a, and miR203 overexpression in chronic ulcers and excisional wounds reduce the capacity of the tissue to regenerate.
Viticchiè et al. 2012 [138]	Original research	In vitro and animal	Skin	miR-203 downregulation in activated keratinocytes during wound closure.
Wang et al. 2012 [139]	Short communication	Animal	Skin	miR-21, miR-31, and miR-203 upregulation improve healing in skin wounds, whereas miR-249 results downregulated 7 days after wounding.
Jin et al. 2013 [140]	Original research	In vitro and animal	Skin	miR-152, miR-365, miR-125a/b-6p. miR-181d, miR-99, miR-100, and miR-30c downregulation in keratinocytes regulates the process of wound healing.
Lin et al. 2013 [141]	Original research	In vitro	Cornea	miR-205 upregulation in corneal epithelial cells stimulates wound healing by inhibiting the KCNJ10 gene.
Li et al. 2015 [142]	Original research	Human	Skin	miR-149, miR-203a, miR-222, and miR-122 downregulation in hypertrophic scar promote wound healing by stimulating cells proliferation and keratinocytes differentiation.
Icli et al. 2016 [143]	Original research	In vitro and animal	Skin	miR-26a overexpression 4 days post-wounding when exposed to high glucose.
Etich et al. 2017 [144]	Original research	Animal	Skin	miR-204 and miR-205 downregulation and miR-31 upregulation in skin wound healing.
Lang et al. 2017 [145]	Original research	In vitro and animal	Skin	miR-149 downregulation in the epidermis improve the quality of collagen deposition in the wound healing process.
Long et al. 2018 [146]	Original research	Animal	Skin	miR-21 upregulation results in an improvement in wound healing in mice.
Lyu et al. 2018 [147]	Original research	Animal	Skin	Upregulation of 19 types of miRNAs and downregulation of 5 types of miRNAs in antemortem burned mice skin.
Ibrahim et al. 2019 [148]	Original research	Animal	Skin	miR-205 and miR-21 upregulation in wound margin, but no statistical significance.
Neri et al. 2019 [149]	Original research	Human	Skin	miR25a-5p, miR128–3p, miR130a-3p, and miR92a-3p overexpression in vital skin lesions and upregulation of miR2–3p and miR150–5p.
Yuan et al., 2019 [150]	Original research	In vitro and animal	Skin	miR-203 downregulation promotes cell proliferation and migration, facilitating the EMT process.
Cao et al. 2020 [151]	Original research	In vitro and animal	Skin	miR-19b role in wound healing by activating the TGF-ß pathway.
He et al. 2020 [152]	Original research	In vitro	Skin	miR-124 role in wound healing by activating Wnt/ß-catenin pathway.
Jiang et al. 2020 [153]	Original research	In vitro	Skin	miR-26a downregulation in keratinocytes promotes cells migration in scratch wound.
Liu et al., 2020 [154]	Original research	Animal	Skin	miR-203 downregulation enhances wound healing and improves healing quality.
Zhang et al. 2020 [155]	Original research	Animal and human	Skin	Overexpression of miR-711 and miR-183–3p in burned skin until 120 h in mouse skins and until 48 h in human burned skin.
Li et al. 2021 [156]	Original research	Human	Skin	miR-19a/b and miR-20a upregulation during wound repair and downregulation in chronic ulcers.

**Table 4:** This table shows the timing of positivity of immunohistoichemical vitality markers in different tissues after wounding. IL, interleukin; α7nAChR, α7 nicotine acetylcholine receptor; A1-ACT, alpha1-antichymotrypsin; RM3/1, anti-CD163 marker; 25F9, mature macrophages marker; GM-CSF, granulocyte macrophage-colony stimulating factor; GPA, glycophorin A; HSP, heat-shock protein; ICAM-1, intercellular adhesion molecule 1; iNOS, inducible nitric oxide synthase; mAb, monoclonal antibody; MCP, monocyte chemoattractant protein; MHC-II, major histocompatibility complex II; MMP, matrix metallopeptidase; RAGE, receptor for advanced glycation endproducts; TNF-α, tumor necrosis factor α; VEGF, vascular endothelial growth factor; VCAM-1, vascular cell adhesion molecule-1; ATP, adenosine triphosphate; MRP, myeloid-related protein; M-CSF, macrophage colony-stimulating factor; MIG, monokine inducible by interferon gamma; ORP, oxygen regulated protein; HLA, human leukocyte antigen; TGF, transforming growth factor; MPO, myeloperoxidases; COX, cyclooxygenase; CB2R, cannabinoid receptor type 2; SP, surfactant protein; HIF, hypoxia inducible factor; AQ, aquaporin; MIP, macrophage inflammatory protein; CML, carboxymethyllysine; Flk, receptor for vascular growth factor; EPC, endothelial progenitor cell; TIMP, metallopeptidase inhibitor; Chil, chitinase-like; INF, interferon; Ub, ubiquitin.

Tissue		Timing of Markers’ Positivity			
	Non Specified	<1 h	<12 h	<24 h	>48 h
**Brain**	-	IL-1ß (1 h), IL-12 p40 (1 h), KC (1 h), LIF (1 h), MIP-2 (1 h)	IL-5 (3 h), IL-6 (3 h), G-CSF (3 h), INF-γ(8 h), CD68 (3 h)	M-CSF (24 h), LN-5 (24 h)	MIB-1 (3–14 days), IL-15 (6 days), IL-18 (6 days), MIG (6 days), IL-12 p70 (1–144 h), TNFα (1–144 h), IL-α (1–144 h), CD68 (10 days), HAM-56 (31 h), 25F9 (100 h)
**Skin**	IL-15, FVIIIra, CD31, CD34, Flk-1, cathepsin D, MHC-II, CD1	Fibronectin (few minutes), selectins (few minutes), IL-1ß (15 min), TNFα (15 min), IL-6 (20 min), defensin (20 min), CD15 (1 h), nonspecific esterases (1 h), tryptase (1 h), chymase (1 h), MRP8 (1 h), MRP14 (1 h), tenascin (1 h), CB2R (1 h), MMP-9 (1 h)	ATPase (2 h), neutrophil elastase (1–3 h), IL-10 (1–3 h), GM-CSF (1–3 h), INF-γ (1–3 h), MPO (1–3 h), COX-2 (1–3 h), collagen I (1–3 h), collagen III (1–3 h), VEGF (1–3 h), VCAM-1 (3 h), ICAM-1 (3–6 h), alkaline phosphatase (3–6 h), MCP-1 (3–6 h), MIP-1α (3–6 h), Ub (3–6 h), iNOS (6 h), IL-6 (6 h), RAGE (6 h), IL-8 (3–12 h)	c-Fos (24 h), c-Jun (24 h), VEGF (24 h), ORP150 (24 h), tenascin (24 h)	AQ3 (3 days), CD3 (3 days), RM3/1 (7 days), 25F9 (11 days), Ki-67 (1–5 days), vimentin (10 days), AQ1 (3–14 days)
**Muscle**	C5b-9, tenascin	Fibronectin (few minutes)	α7nAChR (1–3 h), TIMP-1 (4 h), MMP-9 (12 h)	MMP-2 (6–24 h), TIMP-2 (6–24 h)	TNFα (12–48 h)
**Lung**	SP-A, HIF1-α	HSP27 (<35 min), HSP60 (<35 min), HSP70 (<35 min)	-	-	-
**Kidney**	-	HSP27 (<35 min), HSP60 (<35 min), HSP70 (<35 min)	-	-	-
**Bone**	-	GPA (1 h)	-	-	-

**Table 5:** This table shows the progressive variations of electrolytes and proteins in biological fluid after death. * Mb levels were influenced by putrefactive changes. TNF-α, tumor necrosis factor α; IL, interleukin; Mb, myoglobin; HMGB, high-mobility group box; EPO, erythropoietin; K+, potassium; Na+, sodium; Ca2+, calcium.

Fluid		Timing of Biomarkers and Electrolytes Variations			
	Non Specified	<1 h	<12 h	<24 h	>48 h
**Urine**	-	-	-	Mb (12–24 h)	Mb *
**Serum**	TNFα, IL-1ß, IL-6, IL-10	-	HMGB1 (6–12 h)	-	EPO (7 days)
**Plasma**	Uric acid, ammonia	-	Lactic acid (12 h), hypoxanthine (6–12 h)	-	-
**CFS**	Leucocytic count	Albumin (few minutes)	K+ (12 h), Na+ (6 h), Ca2+ (6 h)	-	-

**Table 6:** This table shows the progressive expression of different mRNAs in time after wounding. Each element depicted in this table is an mRNA. If two or more articles provided contrasting results for the same mRNA, it has not been included. Only mRNAs whose expressions vary, according to a specific timeframe, have been considered. α7nAChR, α7 nicotine acetylcholine receptor; IL, interleukin; MMP9, matrix metallopeptidase; TNF-α, tumor necrosis factor α; TIMP-2, tissue inhibitor of metalloproteinases; MCP-1, monocyte chemoattractant protein 1; MT1-MMP, membrane type-1 matrix metalloproteinases; COX, cyclooxygenase; SNAT2, amino acid transporter 2; CCL, C-C motif chemokine ligand; Fosl1, FOS-like 1; MyOD, myoblast determination protein; FDZ4, frizzled-class receptor 4; SFRP5, secreted frizzled-related protein 5; CSF, colony stimulating factor; PAI1, plasminogen activator inhibitor 1; Pax7, paired-box protein 7; TGF; transforming growth factor; FGF, fibroblast growth factor.

Tissue		Timing of Markers’ Positivity		
	<1 h	<12 h	<24 h	>48 h
**Brain**	FE65 (30 min)	-	-	bFGF (48 h)
**Skin**	IL-10 (15 min), bFGF (1 h), CMA1 (10 min), IL-1ß (30 min), COX-2 (1 h)	IL-6 (6 h), FDZA (12 h)	Fosl1 (24 h), CCL2 (24 h)	IL-1α (2–3 days), TNFα (2–3 days), AFT3 (2 days), CXCL1 (2 days), CXCR2 (2 days), MMP-2 (1–7 days), MMP-9 (3–5 days), CCL3 (5 days), CXCL2 (3 days), CSF2 (3 days), CSF3 (3 days), PAI1 (3 days), DUSP1 (6 days), IL-7 (6 days), VEGFA (6 days), α7nAChR (7 days)
**Muscle**	-	MT1-MMP2 (10 h), TIMP-2 (10 h), MT1-MMP9 (10 h), SNAT2 (4 h)	MyoD (24 h)	MMP-2 (5 days), Pax7 (3–7 days)
**Kidney**	-	-	bFGF (24 h)	-
**Liver**	-	-	bFGF (24 h)	-

**Table 7:** This table summarizes the various miRNAs and their genes and/or proteins target, as studied in the papers included in this review, if available. CCL, chemokine ligand; TGF, transforming growth factor; LepR, leptin receptor; EGR, early growth response; TIMP, metallopeptidase inhibitor; TIAM1, T-cell lymphoma invasion and metastasis inducing protein 1; TP, tumor protein; ITGA, integrin subunit alpha; PI3K, phosphoinositide 3 kinase; AKT, protein kinase B; mTOR, mammalian target of rapamycin; BMP, bone morphogenetic protein; SMAD, small mother against decapentaplegic; GSK, glycogen synthase kinase; IGR1R, insulin-like growth factor 1 receptor; IL, interleukin, PTPRC, protein tyrosine phosphatase receptor type C; CD, differentiation cluster; SHIP2, SH2-containing phosphatidylinositol 3,4,5-trisphosphate 5-phosphatase; RNU6B, RNA U6 small nuclear; MAPK, mitogen-activated protein kinase; NF-kB, nuclear factor kappa-light-chain-enhancer of activated b cells; TCF-4, transcription factor 4; ID-2, inhibitor DNA-binding 2 protein HLH; VEGFA, vascular endothelial growth factor A; NRCAM, neuronal cell adhesion molecule; C-MET, tyrosine-protein kinase Met; LASP1, LIM and SH3 protein 1; RAN, RAs-related nuclear protein; RAPH1, Ras-associated and pleckstrin homology domains-containing protein 1; ERM, ezrin/radixin/moesin; DDK2, dickkopf-related protein 2; FRAT2, FRAT regulator of Wnt signaling pathway 2; MK2, mitogen-activated protein kinase-activated protein kinase 2; YAP1, yes-associated protein 1; MKI67, marker of proliferation Ki-67.

MiRNAs	Target Genes and/or Proteins	References
miR-19-b	CCL1, TGF-ß	[151,155]
miR-21	Factor 3, vinculin, LepR, EGR3, Collagen, TGF-ß, TIMP3, TIAM1, TP53	[136,137,139,145,148]
miR-26a	ITGA5, PI3K/AKT, BMP/SMAD1, GSK3ß	[143,152]
miR-30c	PI3K/AKT, mTOR, IGF1R	[140]
miR-31	IL-1b, PTPRC/CD45, SHIP2, RNU6B, Col1a1	[139,144]
miR-99	PI3K/AKT, mTOR, IGF1R	[140]
miR-100	PI3K/AKT, mTOR, IGF1R	[140]
miR-122	MAPK, insulin signaling pathway	[142]
miR-125a-5p, miR-125b-5p	PI3K/AKT, mTOR, IGF1R	[140]
miR-130a	EGR3	[137]
miR-149	IL-1a, IL-1b, IL-6, TGF-ß, collagen III, NF-kB, RelB, Rel, MAPK	[142,152]
miR-152	PI3K/AKT, mTOR, IGF1R	[140]
miR-181d	PI3K/AKT, mTOR, IGF1R	[140]
miR-184	SHIP2, PI3K/Akt, actin filaments, p-cofilin (via Rho)	[139]
miR-203	MAPK, lysosome, insulin signaling pathway, focal K15, P63, integrin-1, TCF-4, ID-2, CD44, VEGFA, NRCAM, C-MET, Wnt, Notch, Factor 3, vinculin, LepR, EGR3, p63, LASP1, RAN, RAPH1	[137,138,139,142,150,153]
miR-204	IL-1b, PTPRC/CD45, SHIP2, RNU6B, Col1a1	[144]
miR-205	IL-1b, PTPRC/CD45, SHIP2, RNU6B, Col1a1, KCNJ10, SHIP2, PI3K/AKT, actin filaments, p-cofilin (via Rho), p-ERM	[134,141,144,148]
miR-222	DDK2, AXIN2, FRAT2, MAPK	[142]
miR-365	PI3K/AKT, mTOR, IGF1R	[140]
miR-483–3p	MK2, YAP1, ASH2, MKI67	[135]

## Data Availability

Not applicable.

## References

[1] Oehmichen M. (2004). Vitality and time course of wounds. Forensic Sci. Int..

[2] Cappella A., Cattaneo C. (2019). Exiting the limbo of perimortem trauma: A brief review of microscopic markers of hemorrhaging and early healing signs in bone. Forensic Sci. Int..

[3] Byard R.W., Wick R., Gilbert J.D., Donald T. (2008). Histologic dating of bruises in moribund infants and young children. Forensic Sci. Med. Pathol..

[4] Balandiz H., Pehlivan S., Çiçek A.F., Tuğcu H. (2015). Evaluation of Vitality in the Experimental Hanging Model of Rats by Using Immunohistochemical IL-1β Antibody Staining. Am. J. Forensic Med. Pathol..

[5] Turillazzi E., Vacchiano G., Luna-Maldonado A., Neri M., Pomara C., Rabozzi R., Riezzo I., Fineschi V. (2010). Tryptase, CD15 and IL-15 as reliable markers for the determination of soft and hard ligature marks vitality. Histol. Histopathol..

[6] Raekallio J. (1972). Determination of the age of wounds by histochemical and biochemical methods. Forensic Sci..

[7] Bonelli A., Bacci S., Vannelli G., Norelli G. (2003). Immunohistochemical localization of mast cells as a tool for the discrimination of vital and postmortem lesions. Int. J. Leg. Med..

[8] Ishida Y., Kuninaka Y., Nosaka M., Shimada E., Hata S., Yamamoto H., Hashizume Y., Kimura A., Furukawa F., Kondo T. (2018). Forensic application of epidermal AQP3 expression to determination of wound vitality in human compressed neck skin. Int. J. Leg. Med..

[9] Mansueto G., Feola A., Zangani P., Porzio A., Carfora A., Campobasso C.P. (2022). A Clue on the Skin: A Systematic Review on Immunohistochemical Analyses of the Ligature Mark. Int. J. Environ. Res. Public Health.

[10] Grellner W. (2002). Time-dependent immunohistochemical detection of proinflammatory cytokines (IL-1beta, IL-6, TNF-alpha) in human skin wounds. Forensic Sci. Int..

[11] Quan L., Zhu B.-L., Ishikawa T., Michiue T., Zhao D., Li D.-R., Ogawa M., Maeda H. (2008). Postmortem serum erythropoietin levels in establishing the cause of death and survival time at medicolegal autopsy. Int. J. Leg. Med..

[12] Reichelt U., Jung R., Nierhaus A., Tsokos M. (2005). Serial monitoring of interleukin-1beta, soluble interleukin-2 receptor and lipopolysaccharide binding protein levels after death A comparative evaluation of potential postmortem markers of sepsis. Int. J. Leg. Med..

[13] Hougen H.P., Valenzuela A., Lachica E., Villanueva E. (1992). Sudden cardiac death: A comparative study of morphological, histochemical and biochemical methods. Forensic Sci. Int..

[14] Manetti A.C., Maiese A., Baronti A., Mezzetti E., Frati P., Fineschi V., Turillazzi E. (2021). MiRNAs as New Tools in Lesion Vitality Evaluation: A Systematic Review and Their Forensic Applications. Biomedicines.

[15] Manetti A.C., Maiese A., Di Paolo M., De Matteis A., La Russa R., Turillazzi E., Frati P., Fineschi V. (2020). MicroRNAs and Sepsis-Induced Cardiac Dysfunction: A Systematic Review. Int. J. Mol. Sci..

[16] Pinchi E., Frati A., Cantatore S., D’Errico S., La Russa R., Maiese A., Palmieri M., Pesce A., Viola R.V., Fineschi V. (2019). Acute Spinal Cord Injury: A Systematic Review Investigating miRNA Families Involved. Int. J. Mol. Sci..

[17] Zubakov D., Boersma A.W.M., Choi Y., van Kuijk P.F., Wiemer E.A.C., Kayser M. (2010). MicroRNA markers for forensic body fluid identification obtained from microarray screening and quantitative RT-PCR confirmation. Int. J. Leg. Med..

[18] Oono T., Specks U., Eckes B., Majewski S., Hunzelmann N., Timpl R., Krieg T. (1993). Expression of Type VI Collagen mRNA During Wound Healing. J. Investig. Dermatol..

[19] Liberati A., Altman D.G., Tetzlaff J., Mulrow C., Gøtzsche P.C., Ioannidis J.P., Clarke M., Devereaux P.J., Kleijnen J., Moher D. (2009). The PRISMA statement for reporting systematic reviews and meta-analyses of studies that evaluate healthcare interventions: Explanation and elaboration. J. Clin. Epidemiol..

[20] Zhu B.-L., Ishida K., Quan L., Taniguchi M., Oritani S., Kamikodai Y., Fujita M.Q., Maeda H. (2001). Post-mortem urinary myoglobin levels with reference to the causes of death. Forensic Sci. Int..

[21] Mohamed A.A.-R., Elbohi K.M., El Sharkawy N.I., Hassan M.A. (2018). Biochemical and Apoptotic Biomarkers of Experimentally Induced Traumatic Brain Injury: In Relation to Time since Death. Beni-Suef Univ. J. Basic Appl. Sci..

[22] Kobeissy F.H., Shakkour Z., Hayek S.E., Mohamed W., Gold M.S., Wang K.K.W. (2022). Elevation of Pro-inflammatory and Anti-inflammatory Cytokines in Rat Serum after Acute Methamphetamine Treatment and Traumatic Brain Injury. J. Mol. Neurosci..

[23] Njau S.N., Epivatianos P., Tsoukali-Papadopoulou H., Psaroulis D., Stratis J.A. (1991). Magnesium, calcium and zinc fluctuations on skin induced injuries in correlation with time of induction. Forensic Sci. Int..

[24] Chen Y.C., Hu B.J., Yao Q.S., Zhu J.Z. (1995). Diagnostic value of ions as markers for differentiating antemortem from postmortem wounds. Forensic Sci. Int..

[25] He L., Zhu J. (1996). Distinguishing antemortem from postmortem injuries by LTB4 quantification. Forensic Sci. Int..

[26] Laiho K. (1988). Peroxidase activity in traumatic skin lesions. Z. Rechtsmed..

[27] Grellner W., Georg T., Wilske J. (2000). Quantitative analysis of proinflammatory cytokines (IL-1beta, IL-6, TNF-alpha) in human skin wounds. Forensic Sci. Int..

[28] Laiho K. (2004). Albumin as a marker of plasma transudation in experimental skin lesions. Int. J. Leg. Med..

[29] Barnes B.R., Szelenyi E.R., Warren G.L., Urso M.L. (2009). Alterations in mRNA and protein levels of metalloproteinases-2, -9, and -14 and tissue inhibitor of metalloproteinase-2 responses to traumatic skeletal muscle injury. Am. J. Physiol. Physiol..

[30] Kagawa S., Matsuo A., Yagi Y., Ikematsu K., Tsuda R., Nakasono I. (2009). The time-course analysis of gene expression during wound healing in mouse skin. Leg. Med..

[31] Takamiya M., Biwasaka H., Saigusa K., Nakayashiki N., Aoki Y. (2009). Wound age estimation by simultaneous detection of 9 cytokines in human dermal wounds with a multiplex bead-based immunoassay: An estimative method using outsourced examinations. Leg. Med..

[32] Gao T.-L., Yuan X.-T., Yang D., Dai H.-L., Wang W.-J., Peng X., Shao H.-J., Jin Z.-F., Fu Z.-J. (2012). Expression of HMGB1 and RAGE in rat and human brains after traumatic brain injury. J. Trauma Acute Care Surg..

[33] Fan Y.-Y., Zhang S.-T., Yu L.-S., Ye G.-H., Lin K.-Z., Wu S.-Z., Dong M.-W., Han J.-G., Feng X.-P., Li X.-B. (2014). The time-dependent expression of α7nAChR during skeletal muscle wound healing in rats. Int. J. Leg. Med..

[34] Yang S., Sun R., Zhou Z., Zhou J., Liang J., Mu H. (2014). Expression of Amyloid-β Protein and Amyloid-β Precursor Protein After Primary Brain-Stem Injury in Rats. Am. J. Forensic Med. Pathol..

[35] Kimura A., Ishida Y., Nosaka M., Shiraki M., Hama M., Kawaguchi T., Kuninaka Y., Shimada E., Yamamoto H., Takayasu T. (2015). Autophagy in skin wounds: A novel marker for vital reactions. Int. J. Leg. Med..

[36] Birincioğlu I., Akbaba M., Alver A., Kul S., Özer E., Turan N., Şentürk A., Ince I. (2016). Determination of skin wound age by using cytokines as potential markers. J. Forensic Leg. Med..

[37] Tian Z.-L., Jiang S.-K., Zhang M., Wang M., Li J.-Y., Zhao R., Wang L.-L., Li S.-S., Liu M., Zhang M.-Z. (2015). Detection of satellite cells during skeletal muscle wound healing in rats: Time-dependent expressions of Pax7 and MyoD in relation to wound age. Int. J. Leg. Med..

[38] Wang L.-L., Zhao R., Liu C.-S., Liu M., Li S.-S., Li J.-Y., Jiang S.-K., Zhang M., Tian Z.-L., Wang M. (2016). A fundamental study on the dynamics of multiple biomarkers in mouse excisional wounds for wound age estimation. J. Forensic Leg. Med..

[39] Legaz Pérez I., Falcón M., Gimenez M., Diaz F.M., Pérez-Cárceles M.D., Osuna E., Nuno-Vieira D., Luna A. (2017). Diagnosis of vitality in skin wounds in the ligature marks resulting from suicide hanging. Am. J. Forensic Med. Pathol..

[40] He J.-T., Huang H.-Y., Qu D., Xue Y., Zhang K.-K., Xie X.-L., Wang Q. (2018). CXCL1 and CXCR2 as potential markers for vital reactions in skin contusions. Forensic Sci. Med. Pathol..

[41] Qu D., Tan X.-H., Zhang K.-K., Wang Q., Wang H.-J. (2019). ATF3 mRNA, but not BTG2, as a possible marker for vital reaction of skin contusion. Forensic Sci. Int..

[42] Peyron P.-A., Colomb S., Becas D., Adriansen A., Gauchotte G., Tiers L., Marin G., Lehmann S., Baccino E., Delaby C. (2021). Cytokines as new biomarkers of skin wound vitality. Int. J. Leg. Med..

[43] Betz P., Nerlich A., Wilskel J., Tübel J., Wiest I., Penning R., Eisenmenger W. (1992). Immunohistochemical localization of fibronectin as a tool for the age determination of human skin wounds. Int. J. Leg. Med..

[44] Betz P., Nerlich A., Wilske J., Tübel J., Penning R., Eisenmengen W. (1993). The immunohistochemical localization of alpha1-antichymotrypsin and fibronectin and its meaning for the determination of the vitality of human skin wounds. Int. J. Leg. Med..

[45] Fieguth A., Kleemann W.J. (1994). Immunohistochemical examination of skin wounds with antibodies against alpha-1-antichymotrypsin, alpha-2-macroglobulin and lysozyme. Int. J. Leg. Med..

[46] Betz P., Eisenmenger W. (1995). Immunohistochemical analysis of markers for different macrophage phenotypes and their use for a forensic wound age estimation. Int. J. Leg. Med..

[47] Kondo T., Ohshima T. (1996). The dynamics of inflammatory cytokines in the healing process of mouse skin wound: A preliminary study for possible wound age determination. Int. J. Leg. Med..

[48] Dreßler J., Bachmann L., Müller E. (1997). Enhanced expression of ICAM-1 (CD 54) in human skin wounds: Diagnostic value in legal medicine. Agents Actions.

[49] Dressler J., Bachmann L., Kasper M., Hauck J.G., Müller E. (1997). Time dependence of the expression of ICAM-1 (CD 54) in human skin wounds. Int. J. Leg. Med..

[50] Fieguth A., Kleemann W.J., von Wasielewski R., Werner M., Tröger H.D. (1997). Influence of postmortem changes on immunohistochemical reactions in skin. Int. J. Leg. Med..

[51] Dreßler J., Bachmann L., Koch R., Müller E. (1998). Enhanced expression of selectins in human skin wounds. Int. J. Leg. Med..

[52] Grellner W., Dimmeler S., Madea B. (1998). Immunohistochemical detection of fibronectin in postmortem incised wounds of porcine skin. Forensic Sci. Int..

[53] Tabata N. (1998). Morphological changes in traumatized skeletal muscle: The appearance of ‘opaque fibers’ of cervical muscles as evidence of compression of the neck. Forensic Sci. Int..

[54] Dreßler J., Bachmann L., Koch R., Müller E. (1999). Estimation of wound age and VCAM-1 in human skin. Int. J. Leg. Med..

[55] Dressler J., Bachmann L., Strejc P., Koch R., Müller E. (2000). Expression of adhesion molecules in skin wounds: Diagnostic value in legal medicine. Forensic Sci. Int..

[56] Kondo T., Ohshima T., Eisenmenger W. (1999). Immunohistochemical and morphometrical study on the temporal expression of interleukin-1α (IL-1α) in human skin wounds for forensic wound age determination. Int. J. Leg. Med..

[57] Kondo T., Ohshima T., Sato Y., Mayama T., Eisenmenger W. (2000). Immunohistochemical study on the expression of c-Fos and c-Jun in human skin wounds. Histochem. J..

[58] Psaroudakis K., Tzatzarakis M.N., Tsatsakis A.M., Michalodimitrakis M.N. (2001). The application of histochemical methods to the age evaluation of skin wounds: Experimental study in rabbits. Am. J. Forensic Med. Pathol..

[59] Hausmann R., Betz P. (2002). The course of MIB-1 expression by cerebral macrophages following human brain injury. Leg. Med..

[60] Kondo T., Ohshima T., Mori R., Guan D.W., Ohshima K., Eisenmenger W. (2002). Immunohistochemical detection of chemokines in human skin wounds and its application to wound age determination. Int. J. Leg. Med..

[61] Kondo T., Tanaka J., Ishida Y., Mori R., Takayasu T., Ohshima T. (2002). Ubiquitin expression in skin wounds and its application to forensic wound age determination. Int. J. Leg. Med..

[62] Ortiz-Rey J., Suárez-Peñaranda J., Da Silva E., Muñoz-Barús J.I., Miguel-Fraile P.S., De la Fuente-Buceta A., Concheiro-Carro L. (2002). Immunohistochemical detection of fibronectin and tenascin in incised human skin injuries. Forensic Sci. Int..

[63] Suárez-Peñaranda J.M., Rodríguez-Calvo M.S., Ortiz-Rey J.A., Muñoz J.I., Sánchez-Pintos P., Da Silva E.A., De la Fuente-Buceta A., Concheiro-Carro L. (2002). Demonstration of apoptosis in human skin injuries as an indicator of vital reaction. Int. J. Leg. Med..

[64] Bonelli A., Bacci S., Norelli G.A. (2003). Affinity cytochemistry analysis of mast cells in skin lesions: A possible tool to assess the timing of lesions after death. Int. J. Leg. Med..

[65] Fieguth A., Franz D., Lessig R., Kleemann W.J. (2003). Fatal trauma to the neck: Immunohistochemical study of local injuries. Forensic Sci. Int..

[66] Fieguth A., Feldbrügge H., Gerich T., Kleemann W., Tröger H. (2003). The time-dependent expression of fibronectin, MRP8, MRP14 and defensin in surgically treated human skin wounds. Forensic Sci. Int..

[67] Gauchotte G., Wissler M.-P., Casse J.-M., Pujo J., Minetti C., Gisquet H., Vigouroux C., Plénat F., Vignaud J.-M., Martrille L. (2013). FVIIIra, CD15, and tryptase performance in the diagnosis of skin stab wound vitality in forensic pathology. Int. J. Leg. Med..

[68] Ortiz-Rey J.A., Suárez-Peñaranda J.M., Muñoz-Barús J.I., Álvarez C., Miguel P.S., Rodríguez-Calvo M.S., Concheiro-Carro L. (2003). Expression of fibronectin and tenascin as a demonstration of vital reaction in rat skin and muscle. Int. J. Leg. Med..

[69] Hayashi T., Ishida Y., Kimura A., Takayasu T., Eisenmenger W., Kondo T. (2004). Forensic application of VEGF expression to skin wound age determination. Int. J. Leg. Med..

[70] Balažic J., Grajn A., Kralj E., Šerko A., Štefanič B. (2005). Expression of fibronectin suicidal in gunshot wounds. Forensic Sci. Int..

[71] Bacci S., Romagnoli P., Norelli G.A., Forestieri A.L., Bonelli A. (2005). Early increase in TNF-alpha-containing mast cells in skin lesions. Int. J. Leg. Med..

[72] Tarran S., Langlois N.E.I., Dziewulski P., Sztynda T. (2006). Using the Inflammatory Cell Infiltrate to Estimate the Age of Human Burn Wounds: A review and immunohistochemical study. Med. Sci. Law.

[73] Takamiya M., Fujita S., Saigusa K., Aoki Y. (2007). Simultaneous detection of eight cytokines in human dermal wounds with a multiplex bead-based immunoassay for wound age estimation. Int. J. Leg. Med..

[74] Takamiya M., Fujita S., Saigusa K., Aoki Y. (2007). Simultaneous Detections of 27 Cytokines during Cerebral Wound Healing by Multiplexed Bead-Based Immunoassay for Wound Age Estimation. J. Neurotrauma.

[75] Ishida Y., Kimura A., Takayasu T., Eisenmenger W., Kondo T. (2008). Expression of oxygen-regulated protein 150 (ORP150) in skin wound healing and its application for wound age determination. Int. J. Leg. Med..

[76] Ortiz-Rey J.A., Suárez-Peñaranda J.M., San Miguel P., Muñoz J.I., Rodríguez-Calvo M.S., Concheiro L. (2008). Immunohistochemical analysis of P-Selectin as a possible marker of vitality in human cutaneous wounds. J. Forensic Leg. Med..

[77] Neri M., D’Errico S., Fiore C., Pomara C., Rabozzi R., Riezzo I., Turillazzi E., Greco P., Fineschi V. (2009). Stillborn or liveborn? Comparing umbilical cord immunohistochemical expression of vitality markers (tryptase, α1-antichymotrypsin and CD68) by quantitative analysis and confocal laser scanning microscopy. Pathol.-Res. Pract..

[78] Nogami M., Hoshi T., Arai T., Toukairin Y., Takama M., Takahashi I. (2009). Morphology of lymphatic regeneration in rat incision wound healing in comparison with vascular regeneration. Leg. Med..

[79] Oehmichen M., Jakob S., Mann S., Saternus K.S., Pedal I., Meissner C. (2009). Macrophage subsets in mechanical brain injury (MBI)—A contribution to timing of MBI based on immunohistochemical methods: A pilot study. Leg. Med..

[80] Bohnert M., Anderson J., Rothschild M.A., Böhm J. (2010). Immunohistochemical expression of fibronectin in the lungs of fire victims proves intravital reaction in fatal burns. Int. J. Leg. Med..

[81] Cattaneo C., Andreola S., Marinelli E., Poppa P., Porta D., Grandi M. (2010). The detection of microscopic markers of hemorrhaging and wound age on dry bone: A pilot study. Am. J. Forensic Med. Pathol..

[82] Jin J.-Y., Lee S.-H., Yoon H.-J. (2010). A comparative study of wound healing following incision with a scalpel, diode laser or Er,Cr:YSGG laser in guinea pig oral mucosa: A histological and immunohistochemical analysis. Acta Odontol. Scand..

[83] Guler H., Aktas E.O., Karali H., Aktas S. (2011). The Importance of Tenascin and Ubiquitin in Estimation of Wound Age. Am. J. Forensic Med. Pathol..

[84] Ryu S.-W., Lee S.-H., Yoon H.-J. (2011). A comparative histological and immunohistochemical study of wound healing following incision with a scalpel, CO_2_laser or Er,Cr:YSGG laser in the Guinea pig oral mucosa. Acta Odontol. Scand..

[85] Taborelli A., Andreola S., Di Giancamillo A., Gentile G., Domeneghini C., Grandi M., Cattaneo C. (2011). The use of the anti-Glycophorin a antibody in the detection of red blood cell residues in human soft tissue lesions decomposed in air and water: A pilot study. Med. Sci. Law.

[86] Capatina C.O., Ceausu M., Curca G.C., Tabirca D.D., Hostiuc S. (2012). Immunophenotypical expression of adhesion molecules in vital reaction. Rom. J. Leg. Med..

[87] Cecchi R., Aromatario M., Frati P., Lucidi D., Ciallella C. (2012). Death due to crush injuries in a compactor truck: Vitality assessment by immunohistochemistry. Int. J. Leg. Med..

[88] Ishida Y., Kimura A., Nosaka M., Kuninaka Y., Takayasu T., Eisenmenger W., Kondo T. (2012). Immunohistochemical analysis on cyclooxygenase-2 for wound age determination. Int. J. Leg. Med..

[89] Zheng J.-L., Yu T.-S., Li X.-N., Fan Y.-Y., Ma W.-X., Du Y., Zhao R., Guan D.-W. (2012). Cannabinoid receptor type 2 is time-dependently expressed during skin wound healing in mice. Int. J. Leg. Med..

[90] Capatina C., Ceausu M., Hostiuc S. (2013). Usefulness of Fibronectin and P-selectin as markers for vital reaction in uncontrolled conditions. Rom. J. Leg. Med..

[91] Agha A. (2013). Histological study and immunohistochemical expression of inducible nitric oxide synthase and vascular endothelial growth factor in skin wound healing and its application for forensic wound age determination. Al-Azhar J. Pharm. Sci..

[92] Akbaba M., Kara S., Demir T., Temizer M., Dulger H., Bakir K. (2014). Immunohistochemical determination of wound age in mice. Gaziantep Med. J..

[93] Bacci S., Defraia B., Cinci L., Calosi L., Guasti D., Pieri L., Lotti V., Bonelli A., Romagnoli P. (2014). Immunohistochemical analysis of dendritic cells in skin lesions: Correlations with survival time. Forensic Sci. Int..

[94] Cecchi R., Sestili C., Prosperini G., Cecchetto G., Vicini E., Viel G., Muciaccia B. (2013). Markers of mechanical asphyxia: Immunohistochemical study on autoptic lung tissues. Int. J. Leg. Med..

[95] Kubo H., Hayashi T., Ago K., Ago M., Kanekura T., Ogata M. (2014). Forensic diagnosis of ante- and postmortem burn based on aquaporin-3 gene expression in the skin. Leg. Med..

[96] Montisci M., Corradin M., Giacomelli L., Viel G., Cecchetto G., Ferrara S.D. (2014). Can immunohistochemistry quantification of Cathepsin-D be useful in the differential diagnosis between vital and post-mortem wounds in humans?. Med. Sci. Law.

[97] van de Goot F.R., Korkmaz H.I., Fronczek J., Witte B.I., Visser R., Ulrich M.M., Begieneman M.P., Rozendaal L., Krijnen P.A., Niessen H.W. (2014). A new method to determine wound age in early vital skin injuries: A probability scoring system using expression levels of Fibronectin, CD62p and Factor VIII in wound hemorrhage. Forensic Sci. Int..

[98] Capatina C.O., Chirica V.I., Martius E., Isaila O.M., Ceausu M. (2015). Are P-selectin and fibronectin truly useful for the vital reaction? Case presentation. Rom. J. Leg. Med..

[99] El Deeb N.M.F., Badr El Dine F.M. (2015). Evaluation of lymphatic regeneration in rat incisional wound healing and its use in wound age estimation. Alex. J. Med..

[100] Fronczek J., Lulf R., Korkmaz H.I., Witte B.I., van de Goot F.R., Begieneman M.P., Schalkwijk C., Krijnen P.A., Rozendaal L., Niessen H.W. (2015). Analysis of inflammatory cells and mediators in skin wound biopsies to determine wound age in living subjects in forensic medicine. Forensic Sci. Int..

[101] Ishida Y., Kimura A., Nosaka M., Kuninaka Y., Shimada E., Yamamoto H., Nishiyama K., Inaka S., Takayasu T., Eisenmenger W. (2015). Detection of endothelial progenitor cells in human skin wounds and its application for wound age determination. Int. J. Leg. Med..

[102] Kara1 S., Akbaba M., Kul S., Bakır K. (2016). Is it possible to make early wound age estimation by immunohistochemical methods?. Rom. J. Leg. Med..

[103] Yu T.S., Li Z., Zhao R., Zhang Y., Zhang Z.-H., Guan D.W. (2016). Time-dependent Expression of MMP-2 and TIMP-2 after Rats Skeletal Muscle Contusion and Their Application to Determine Wound Age. J. Forensic Sci..

[104] Abo El-Noor M.M., Elgazzar F.M., Alshenawy H.A. (2017). Role of inducible nitric oxide synthase and interleukin-6 expression in estimation of skin burn age and vitality. J. Forensic Leg. Med..

[105] Ji X.-Y., Chen Y., Ye G.-H., Dong M.-W., Lin K.-Z., Han J.-G., Feng X.-P., Li X.-B., Yu L.-S., Fan Y.-Y. (2017). Detection of RAGE expression and its application to diabetic wound age estimation. Int. J. Leg. Med..

[106] Legaz I., Pérez-Cárceles M.D., Gimenez M., Martínez-Díaz F., Osuna E., Luna A. (2018). Immunohistochemistry as a tool to characterize human skin wounds of hanging marks. Rom. J. Leg. Med..

[107] Murase T., Yamamoto T., Koide A., Yagi Y., Kagawa S., Tsuruya S., Abe Y., Umehara T., Ikematsu K. (2017). Temporal expression of chitinase-like 3 in wounded murine skin. Rom. J. Leg. Med..

[108] Doberentz E., Madea B. (2018). Supravital expression of heat-shock proteins. Forensic Sci. Int..

[109] Ishida Y., Kuninaka Y., Furukawa F., Kimura A., Nosaka M., Fukami M., Yamamoto H.A., Kato T., Shimada E., Hata S. (2017). Immunohistochemical analysis on aquaporin-1 and aquaporin-3 in skin wounds from the aspects of wound age determination. Forensic Sci. Int..

[110] Metwally E.S., Madboly A.G., Farag A., Abdelaziz T.A., Farag H.A. (2018). Reliability of Fibronectin and P-selectin as Indicators of Vitality and Age of Wounds: An Immunohistochemical Study on Human Skin Wounds. Mansoura J. Forensic Med. Clin. Toxicol..

[111] De Matteis A., dell’Aquila M., Maiese A., Frati P., La Russa R., Bolino G., Fineschi V. (2019). The Troponin-I fast skeletal muscle is reliable marker for the determination of vitality in the suicide hanging. Forensic Sci. Int..

[112] Focardi M., Puliti E., Grifoni R., Palandri M., Bugelli V., Pinchi V., Norelli G.A., Bacci S. (2019). Immunohistochemical localization of Langerhans cells as a tool for vitality in hanging mark wounds: A pilot study. Aust. J. Forensic Sci..

[113] Khalaf A.A., Hassanen E.I., Zaki A.R., Tohamy A.F., Ibrahim M.A. (2019). Histopathological, immunohistochemical, and molecular studies for determination of wound age and vitality in rats. Int. Wound J..

[114] Focardi M., Bugelli V., Venturini M., Bianchi I., Defraia B., Pinchi V., Bacci S. (2020). Increased expression of iNOS by Langerhans cells in hanging marks. Aust. J. Forensic Sci..

[115] Maiese A., De Matteis A., Bolino G., Turillazzi E., Frati P., Fineschi V. (2020). Hypo-Expression of Flice-Inhibitory Protein and Activation of the Caspase-8 Apoptotic Pathways in the Death-Inducing Signaling Complex Due to Ischemia Induced by the Compression of the Asphyxiogenic Tool on the Skin in Hanging Cases. Diagnostics.

[116] Baldari B., Vittorio S., Sessa F., Cipolloni L., Bertozzi G., Neri M., Cantatore S., Fineschi V., Aromatario M. (2021). Forensic Application of Monoclonal Anti-Human Glycophorin A Antibody in Samples from Decomposed Bodies to Establish Vitality of the Injuries. A Preliminary Experimental Study. Healthcare.

[117] Bertozzi G., Ferrara M., La Russa R., Pollice G., Gurgoglione G., Frisoni P., Alfieri L., De Simone S., Neri M., Cipolloni L. (2021). Wound Vitality in Decomposed Bodies: New Frontiers Through Immunohistochemistry. Front. Med..

[118] Niedecker A., Huhn R., Ritz-Timme S., Mayer F. (2021). Complex challenges of estimating the age and vitality of muscle wounds: A study with matrix metalloproteinases and their inhibitors on animal and human tissue samples. Int. J. Leg. Med..

[119] Prangenberg J., Doberentz E., Witte A.-L., Madea B. (2021). Aquaporin 1 and 3 as local vitality markers in mechanical and thermal skin injuries. Int. J. Leg. Med..

[120] Wegner A., Doberentz E., Madea B. (2021). Death in the sauna-vitality markers for heat exposure. Int. J. Leg. Med..

[121] Khismatullin R.R., Shakirova A.Z., Weisel J.W., Litvinov R.I. (2020). Age-dependent Differential Staining of Fibrin in Blood Clots and Thrombi. BioNanoScience.

[122] Zamboni P., De Mattei M., Ongaro A., Fogato L., Carandina S., De Palma M., Tognazzo S., Scapoli G.L., Serino M.L., Caruso A. (2004). Factor XIII Contrasts the Effects of Metalloproteinases in Human Dermal Fibroblast Cultured Cells. Vasc. Endovascular Surg..

[123] Ohshima T., Sato Y. (1998). Time-dependent expression of interleukin-10 (IL-10) mRNA during the early phase of skin wound healing as a possible indicator of wound vitality. Int. J. Leg. Med..

[124] Sato Y., Ohshima T. (2000). The expression of mRNA of proinflammatory cytokines during skin wound healing in mice: A preliminary study for forensic wound age estimation (II). Int. J. Leg. Med..

[125] Iino M., Nakatome M., Ogura Y., Fujimura H., Kuroki H., Inoue H., Ino Y., Fujii T., Terao T., Matoba R. (2003). Real-time PCR quantitation of FE65 a beta-amyloid precursor protein-binding protein after traumatic brain injury in rats. Int. J. Leg. Med..

[126] Takamiya M., Saigusa K., Nakayashiki N., Aoki Y. (2003). Studies on mRNA expression of basic fibroblast growth factor in wound healing for wound age determination. Int. J. Leg. Med..

[127] Bai R., Wan L., Shi M. (2008). The time-dependent expressions of IL-1beta, COX-2, MCP-1 mRNA in skin wounds of rabbits. Forensic Sci. Int..

[128] Sun J.-H., Wang Y.-Y., Zhang L., Gao C.-R., Zhang L.-Z., Guo Z. (2009). Time-dependent expression of skeletal muscle troponin I mRNA in the contused skeletal muscle of rats: A possible marker for wound age estimation. Int. J. Leg. Med..

[129] Sun J.-H., Nan L.-H., Gao C.-R., Wang Y.-Y. (2011). Validation of reference genes for estimating wound age in contused rat skeletal muscle by quantitative real-time PCR. Int. J. Leg. Med..

[130] Du Q.-X., Sun J.-H., Zhang L.-Y., Liang X.-H., Guo X.-J., Gao C.-R., Wang Y.-Y. (2013). Time-dependent expression of SNAT2 mRNA in the contused skeletal muscle of rats: A possible marker for wound age estimation. Forensic Sci. Med. Pathol..

[131] Palagummi S., Harbison S., Fleming R. (2013). A time-course analysis of mRNA expression during injury healing in human dermal injuries. Int. J. Leg. Med..

[132] Kameyama H., Udagawa O., Hoshi T., Toukairin Y., Arai T., Nogami M. (2015). The mRNA expressions and immunohistochemistry of factors involved in angiogenesis and lymphangiogenesis in the early stage of rat skin incision wounds. Leg. Med..

[133] Zhu X.-Y., Du Q.-X., Li S.-Q., Sun J.-H. (2016). Comparison of the homogeneity of mRNAs encoding SFRP5, FZD4, and Fosl1 in post-injury intervals: Subcellular localization of markers may influence wound age estimation. J. Forensic Leg. Med..

[134] Ye M.-Y., Xu D., Liu J.-C., Lyu H.-P., Xue Y., He J.-T., Huang H.-Y., Zhang K.-K., Xie X.-L., Wang Q. (2018). IL-6 and IL-20 as potential markers for vitality of skin contusion. J. Forensic Leg. Med..

[135] Du Q.-X., Li N., Dang L.-H., Dong T.-N., Lu H.-L., Shi F.-X., Jin Q.-Q., Jie C., Sun J.-H. (2019). Temporal expression of wound healing–related genes inform wound age estimation in rats after a skeletal muscle contusion: A multivariate statistical model analysis. Int. J. Leg. Med..

[136] Yu J., Peng H., Ruan Q., Fatima A., Getsios S., Lavker R.M. (2010). MicroRNA-205 promotes keratinocyte migration via the lipid phosphatase SHIP2. FASEB J..

[137] Bertero T., Gastaldi C., Bourget-Ponzio I., Imbert V., Loubat A., Selva E., Busca R., Mari B., Hofman P., Barbry P. (2011). miR-483-3p controls proliferation in wounded epithelial cells. FASEB J..

[138] Yang X., Wang J., Guo S.-L., Fan K.-J., Li J., Wang Y.-L., Teng Y., Yang X. (2013). miR-21 Promotes Keratinocyte Migration and Re-epithelialization during Wound Healing. Int. J. Biol. Sci..

[139] Pastar I., Khan A.A., Stojadinovic O., Lebrun E.A., Medina M.C., Brem H., Kirsner R.S., Jimenez J.J., Leslie C., Tomic-Canic M. (2012). Induction of Specific MicroRNAs Inhibits Cutaneous Wound Healing. J. Biol. Chem..

[140] Viticchiè G., Lena A.M., Cianfarani F., Odorisio T., Annicchiarico-Petruzzelli M., Melino G., Candi E. (2012). MicroRNA-203 contributes to skin re-epithelialization. Cell Death Dis..

[141] Wang T., Feng Y., Sun H., Zhang L., Hao L., Shi C., Wang J., Li R., Ran X., Su Y. (2012). miR-21 Regulates Skin Wound Healing by Targeting Multiple Aspects of the Healing Process. Am. J. Pathol..

[142] Jin Y., Tymen S.D., Chen D., Fang Z.J., Zhao Y., Dragas D., Dai Y., Marucha P.T., Zhou X. (2013). MicroRNA-99 Family Targets AKT/mTOR Signaling Pathway in Dermal Wound Healing. PLoS ONE.

[143] Lin D., Halilovic A., Yue P., Bellner L., Wang K., Wang L., Zhang C. (2013). Inhibition of miR-205 Impairs the Wound-Healing Process in Human Corneal Epithelial Cells by Targeting KIR4.1 (KCNJ10). Investig. Opthalmol. Vis. Sci..

[144] Li P., He Q., Luo C., Qian L. (2015). Differentially expressed miRNAs in acute wound healing of the skin: A pilot study. Medicine.

[145] Icli B., Nabzdyk C.S., Lujan-Hernandez J., Cahill M., Auster M.E., Wara A., Sun X., Ozdemir D., Giatsidis G., Orgill D.P. (2016). Regulation of impaired angiogenesis in diabetic dermal wound healing by microRNA-26a. J. Mol. Cell. Cardiol..

[146] Etich J., Bergmeier V., Pitzler L., Brachvogel B. (2016). Identification of a reference gene for the quantification of mRNA and miRNA expression during skin wound healing. Connect. Tissue Res..

[147] Lang H., Zhao F., Zhang T., Liu X., Wang Z., Wang R., Shi P., Pang X. (2017). MicroRNA-149 contributes to scarless wound healing by attenuating inflammatory response. Mol. Med. Rep..

[148] Long S., Zhao N., Ge L., Wang G., Ran X., Wang J., Su Y., Wang T. (2018). MiR-21 ameliorates age-associated skin wound healing defects in mice. J. Gene Med..

[149] Lyu H.-P., Cheng M., Liu J.-C., Ye M.-Y., Xu D., He J.-T., Xie X.-L., Wang Q. (2018). Differentially expressed microRNAs potential markers for vital reaction of burned skin. J. Forensic Med..

[150] Ibrahim S.F., Ali M.M., Basyouni H., Rashed L.A., Amer E.A.E., El-Kareem D.A. (2019). Histological and miRNAs postmortem changes in incisional wound. Egypt. J. Forensic Sci..

[151] Neri M., Fabbri M., D’Errico S., Di Paolo M., Frati P., Gaudio R.M., La Russa R., Maiese A., Marti M., Pinchi E. (2019). Regulation of miRNAs as new tool for cutaneous vitality lesions demonstration in ligature marks in deaths by hanging. Sci. Rep..

[152] Yuan L., Sun Y., Xu M., Zeng F., Xiong X. (2019). miR-203 Acts as an Inhibitor for Epithelial-Mesenchymal Transition Process in Diabetic Foot Ulcers via Targeting Interleukin-8. Neuroimmunomodulation.

[153] Cao G., Chen B., Zhang X., Chen H. (2020). Human Adipose-Derived Mesenchymal Stem Cells-Derived Exosomal microRNA-19b Promotes the Healing of Skin Wounds Through Modulation of the CCL1/TGF-β Signaling Axis. Clin. Cosmet. Investig. Dermatol. Biosci. Rep..

[154] He L., Zhu C., Jia J., Hao X.-Y., Yu X.-Y., Liu X.-Y., Shu M.-G. (2020). ADSC-Exos containing MALAT1 promotes wound healing by targeting miR-124 through activating Wnt/β-catenin pathway. Biosci. Rep..

[155] Jiang Z., Wei J., Yang W., Li W., Liu F., Yan X., Yan X., Hu N., Li J. (2020). MicroRNA-26a inhibits wound healing through decreased keratinocytes migration by regulating ITGA5 through PI3K/AKT signaling pathway. Biosci. Rep..

[156] Liu J., Shu B., Zhou Z., Xu Y., Liu Y., Wang P., Xiong K., Xie J. (2020). Involvement of miRNA203 in the proliferation of epidermal stem cells during the process of DM chronic wound healing through Wnt signal pathways. Stem Cell Res. Ther..

[157] Zhang K., Cheng M., Xu J., Chen L., Li J., Li Q., Xie X., Wang Q. (2020). MiR-711 and miR-183-3p as potential markers for vital reaction of burned skin. Forensic Sci. Res..

[158] Li D., Peng H., Qu L., Sommar P., Wang A., Chu T., Li X., Bi X., Liu Q., Sérézal I.G. (2020). miR-19a/b and miR-20a Promote Wound Healing by Regulating the Inflammatory Response of Keratinocytes. J. Investig. Dermatol..

[159] MacNeil T., Vathiotis I.A., Martinez-Morilla S., Yaghoobi V., Zugazagoitia J., Liu Y., Rimm D.L. (2020). Antibody validation for protein expression on tissue slides: A protocol for immunohistochemistry. BioTechniques.

[160] Gambella A., Porro L., Pigozzi S., Fiocca R., Grillo F., Mastracci L. (2017). Section detachment in immunohistochemistry: Causes, troubleshooting, and problem-solving. Histochem. Cell Biol..

[161] Choudhury K.R., Yagle K.J., Swanson P.E., Krohn K.A., Rajendran J.G. (2009). A Robust Automated Measure of Average Antibody Staining in Immunohistochemistry Images. J. Histochem. Cytochem..

[162] Rizzardi A.E., Johnson A.T., Vogel R.I., Pambuccian S.E., Henriksen J., Skubitz A.P., Metzger G.J., Schmechel S.C. (2012). Quantitative comparison of immunohistochemical staining measured by digital image analysis versus pathologist visual scoring. Diagn. Pathol..

[163] Konsti J., Lundin M., Linder N., Haglund C., Blomqvist C., Nevanlinna H., Aaltonen K., Nordling S., Lundin J. (2012). Effect of image compression and scaling on automated scoring of immunohistochemical stainings and segmentation of tumor epithelium. Diagn. Pathol..

[164] Asimaki A., Tandri H., Huang H., Halushka M.K., Gautam S., Basso C., Thiene G., Tsatsopoulou A., Protonotarios N., McKenna W.J. (2009). A New Diagnostic Test for Arrhythmogenic Right Ventricular Cardiomyopathy. N. Engl. J. Med..

[165] Kemp W.L. (2016). Postmortem Change and its Effect on Evaluation of Fractures. Acad. Forensic Pathol..

[166] Wheatley B.P. (2008). Perimortem or Postmortem Bone Fractures? An Experimental Study of Fracture Patterns in Deer Femora. J. Forensic Sci..

[167] Grellner W., Madea B. (2007). Demands on scientific studies: Vitality of wounds and wound age estimation. Forensic Sci. Int..

[168] Dettmeyer R.B., Verhoff M.A., Schutz H.F., Dettmeyer R.B., Verhoff M.A., Schutz H.F. (2014). Vital reactions. Forensic Medicine.

[169] Taboubi S., Milanini J., Delamarre E., Parat F., Garrouste F., Pommier G., Takasaki J., Hubaud J., Kovacic H., Lehmann M. (2007). Galpha(q/11)-coupled P2Y2 nucleotide receptor inhibits human keratinocyte spreading and migration. FASEB J..

[170] Kakinuma N., Roy B.C., Zhu Y., Wang Y., Kiyama R. (2008). Kank regulates RhoA-dependent formation of actin stress fibers and cell migration via 14-3-3 in PI3K-Akt signaling. J. Cell Biol..

[171] Squarize C.H., Castilho R.M., Bugge T.H., Gutkind J.S. (2010). Accelerated Wound Healing by mTOR Activation in Genetically Defined Mouse Models. PLoS ONE.

[172] Whyte J.L., Smith A.A., Helms J.A. (2012). Wnt Signaling and Injury Repair. Cold Spring Harb. Perspect. Biol..

[173] Zhang S., Ishida Y., Ishigami A., Nosaka M., Kuninaka Y., Hata S., Yamamoto H., Hashizume Y., Matsuki J., Yasuda H. (2022). Forensic Application of Epidermal Ubiquitin Expression to Determination of Wound Vitality in Human Compressed Neck Skin. Front. Med..

[174] Ishida Y., Nosaka M., Kondo T. (2022). Bone Marrow-Derived Cells and Wound Age Estimation. Front. Med..

[175] Ros A.C., Bacci S., Luna A., Legaz I. (2022). Forensic Impact of the Omics Science Involved in the Wound: A Systematic Review. Front. Med..

[176] Gitto L., Bonaccorso L., Maiese A., dell’Aquila M., Arena V., Bolino G. (2015). A scream from the past: A multidisciplinary approach in a concealment of a corpse found mummified. J. Forensic Leg. Med..

[177] Prangenberg J., Doberentz E., Madea B. (2021). Mini Review: Forensic Value of Aquaporines. Front. Med..

[178] Casse J.M., Martrille L., Vignaud J.M., Gauchotte G. (2016). Skin Wounds Vitality Markers in Forensic Pathology: An Updated Review. Med. Sci. Law..

[179] Zamboni P., Gemmati D. (2007). Clinical Implications of Gene Polymorphisms in Venous Leg Ulcer: A Model in Tissue Injury and Reparative Process. Thromb. Haemost..

[180] Maiese A., Del Duca F., Santoro P., Pellegrini L., De Matteis A., La Russa R., Frati P., Fineschi V. (2022). An Overview on Actual Knowledge About Immunohistochemical and Molecular Features of Vitality, Focusing on the Growing Evidence and Analysis to Distinguish Between Suicidal and Simulated Hanging. Front. Med. (Lausanne).

[181] Dell’aquila M., Maiese A., De Matteis A., Viola R.V., Arcangeli M., La Russa R., Fineschi V. (2021). Traumatic brain injury: Estimate of the age of the injury based on neuroinflammation, endothelial activation markers and adhesion molecules. Histol. Histopathol..

